# Characteristics of children with a psychiatric disorder in 1999, 2004 and 2017: an analysis of the national child mental health surveys of England

**DOI:** 10.1111/jcpp.14040

**Published:** 2024-07-24

**Authors:** Jessica M. Armitage, Tamsin Newlove‐Delgado, Tamsin Ford, Sally McManus, Stephan Collishaw

**Affiliations:** ^1^ Wolfson Centre for Young People's Mental Health Cardiff University Cardiff UK; ^2^ Division of Psychological Medicine and Clinical Neurosciences, MRC Centre for Neuropsychiatric Genetics and Genomics, Cardiff University School of Medicine Cardiff University Cardiff UK; ^3^ Medical School University of Exeter Exeter UK; ^4^ Department of Psychiatry Cambridge University Cambridge UK; ^5^ Health and Social Care National Centre for Social Research London UK; ^6^ School of Health and Psychological Sciences City, University of London London UK

**Keywords:** Child mental health, psychiatric disorder, secular change, time trends, functioning

## Abstract

**Background:**

While research has described the profile of children with poor mental health, little is known about whether this profile and their needs have changed over time. Our aim was to investigate whether levels of difficulties and functional impact faced by children with a psychiatric disorder have changed over time, and whether sociodemographic and family correlates have changed.

**Methods:**

Samples were three national probability surveys undertaken in England in 1999, 2004 and 2017 including children aged 5–15 years. Psychiatric disorders were assessed using the Development and Well‐Being Assessment (DAWBA), a standardised multi‐informant diagnostic tool based on the tenth International Classification of Diseases (ICD‐10). The impact and difficulties of having a disorder (emotional, behavioural or hyperkinetic) were compared over time using total difficulty and impact scores from the Strengths and Difficulties Questionnaire (SDQ). Analyses explored the impact of having any disorder, as well as for each disorder separately. Regression analyses compared associations between disorders and sociodemographic factors over time.

**Results:**

Parent‐ and adolescent‐reported total SDQ difficulty and impact scores increased between 1999 and 2017 for children and adolescents with disorders. No differences were noted when using teacher ratings. No differences in total SDQ difficulty score were found for children without a disorder. Comparison of sociodemographic correlates across the surveys over time revealed that ethnic minority status, living in rented accommodation and being in the lowest income quintile had a weaker association with disorder in 2017 compared to 1999.

**Conclusions:**

Our study reveals a concerning trend; children with a disorder in 2017 experienced more severe difficulties and greater impact on functioning at school, home and in their daily lives, compared to children with a disorder in earlier decades. Research is needed to identify and understand factors that may explain the changing nature and level of need among children with a disorder.

## Background

Child and adolescent psychiatric disorders are common. A recent meta‐analysis estimated a prevalence of 12.7% in 4–18 year olds (Barican et al., 2022), and in England, these problems are increasing (Sadler et al., 2018). Even prior to the Covid‐19 pandemic, international evidence suggested a rise in emotional problems. This increase is concerning not only because of the distress for children, young people and their families, but also due to the range of adverse outcomes and associated societal costs linked with mental ill health in children, including an increased risk for adult mental health conditions, impaired social relationships, educational exclusion and underachievement, and poorer physical health (Costello & Maughan, 2015).

However, while many studies have examined trends in prevalence of child and adolescent mental health problems, less is known about trends in symptom severity, functional implications or sociodemographic correlates of child and adolescent mental health disorders. This is an important question and provides necessary information to providers and planners of mental health services. If patients presenting to them have greater or more complex needs, they should be aware of this. In addition, changes in the impact of disorder or difficulties with functioning over time may alert us to changes in the context and environment in which children are growing up and hence suggest targets for public health interventions. Increased impact and poorer functioning in childhood also predict poorer outcomes (Stringaris & Goodman, 2013), and hence an understanding of trends in the functioning of children with disorder can provide insights into future outcomes on a population level. One of the few studies in this area (Sellers, Maughan, Pickles, Thapar, & Collishaw, 2015) reported increases in impact scores between 1999 and 2008 for children scoring in the abnormal range on the Strengths and Difficulties Questionnaire (SDQ), finding an increase in the impact of the child's problems on domains such as learning, leisure and friendships. In another study, Sellers et al. (2019) found stronger associations between child mental health problems and longer term poorer social and academic functioning among cohorts born more recently, which suggests that the consequences of having a mental health problem may have worsened over time.

Evidence from epidemiological studies has also demonstrated considerable specificity, with increasing trends more marked for emotional problems and for females than males (Armitage et al., 2023; Blomqvist, Henje Blom, Hägglöf, & Hammarström, 2019; Collishaw, 2015). Some research also suggests that certain sociodemographic factors, including housing tenure and low income, may have become more strongly associated with emotional difficulties in more recent generations (Langton, Collishaw, Goodman, Pickles, & Maughan, 2011; McElroy, Tibber, Fearon, Patalay, & Ploubidis, 2023). However, much of the epidemiological research to date has been based on parent‐reported problems identified using symptom screens rather than diagnostic assessments (Langton et al., 2011; McElroy et al., 2023). Examining whether the characteristics and correlates of children and adolescents meeting criteria for a psychiatric disorder have changed over time is, therefore, necessary to extend existing research to further inform current population health priorities and target efforts at prevention.

The current study represents the first to use unique data from the three national child mental health surveys undertaken in England in 1999, 2004 and 2017 to test for changes in the level of difficulties reported between these surveys and whether the characteristics of children with a psychiatric disorder have changed over time. The samples are part of a series of population‐based probability surveys, including identical multi‐informant and clinically validated assessments of psychiatric disorder. As such, they allow comparison of the profiles of children meeting diagnostic criteria for International Classification of Diseases (ICD‐10) psychiatric disorders in a community sample across all three time points (World Health Organization, 1993).

The aims of our study were:

1. To investigate whether the degree of difficulty and impact in children with a psychiatric disorder has changed over time.
2. To test whether associations between the risk of disorder and demographic, socioeconomic and family factors have changed across the three surveys.

## Methods

### Samples

Samples were drawn from the British Child and Adolescent Mental Health Surveys (BCAMHS) 1999 and 2004 and the latest in the series, the Mental Health of Children and Young People in England (MHCYP) 2017 survey (Ford et al., 2020). The surveys were conducted in 1999 (ages 5–15 years), 2004 (ages 5–16 years) and 2017 (ages 2–19 years), with the 1999 and 2004 samples drawn from the Child Benefit Register, which had universal coverage. The 2017 sample was drawn from the National Health Service (NHS) Patient Register using stratified, multistage, random probability sampling as Child Benefit was no longer universal. The 1999 and 2004 samples spanned Great Britain (England, Wales and Scotland), but the 2017 sample included England only. To increase comparability between the three surveys, we restrict our analyses to children aged 5–15 years in England.

Across the 1999 and 2004 surveys, approximately 10% of postal sectors were sampled with a probability related to size of sector, and in 2017, children were selected from a stratified probability sample of approximately 6.3% of postcode sectors (Ford et al., 2020). The 1999 and 2004 samples were stratified by regional health authority and socioeconomic group within it, which represented approximately 90% of all British children once adjusted for inaccurate or ineligible benefit records (Ford et al., 2020). For the 2017 sample, these were stratified by Government office region, selected relative to the size of the region and sorted on factors associated with mental health disorders.

Mental health assessments were completed with 73% and 65% of the original 1999 and 2004 samples, respectively, which equated to 83% and 76% of those approached for interview. For the 2017 sample, mental health assessments were conducted with 52% of those eligible and 59% of those approached.

Data were collected from parents (94% from the biological mothers), children (aged ≥ 11 years) and teachers (the family nominated the teacher who knew the child best) across all three surveys (see Table S1 for further information). Parents and children were interviewed face‐to‐face at home by trained lay interviewers using computer‐assisted interviews, with self‐reported questionnaires for sensitive topics. Teachers responded to a mailed questionnaire if parents consented. The characteristics of the overall 1999 sample were broadly similar to those of the 2017 sample (see Tables S2 and S3). Notable differences included an approximate doubling of the proportion of children from ethnic minority backgrounds and a tripling in the proportion living in rented accommodation between 1999 and 2017.

These secondary analyses were approved by the University of Exeter Medical School REC (Nov20/D/270). The MHCYP 2017 data were used under a data sharing agreement with NHS England (DARS‐NIC‐424336‐T7K7T‐).

### Measures

#### ICD‐10 psychiatric disorders

Psychiatric disorders were assigned across all three samples using the validated Development and Well‐Being Assessment (DAWBA) (Goodman, Ford, Simmons, Gatward, & Meltzer, 2000). This tool combines structured and semi‐structured questions about psychiatric disorders, based on the ICD‐10 criteria (World Health Organization, 1993). Within all three surveys, the DAWBA incorporated information from parents and children from interviews, and from teacher questionnaires where the parent consented. Computer‐generated predictions of disorders were produced using algorithms based on diagnostic criteria and reviewed alongside the free text responses by trained clinicians who assigned final diagnoses based on ICD‐10 criteria. Further information about the procedures for diagnosis in the DAWBA are reported elsewhere (see Ford, Goodman, & Meltzer, 2003), and downloadable versions of the measures are available at https://dawba.info/.

For our main analyses, we identified individuals meeting the criteria for at least one ICD‐10 psychiatric disorder, including anxiety disorders, depressive disorders, conduct disorders (including Oppositional Defiant Disorder (ODD)), hyperkinetic disorders, eating disorders and tic disorders. Sensitivity analyses then included individuals with either an emotional disorder (anxiety or depressive disorder), a behavioural disorder (conduct disorder or ODD), a hyperkinetic disorder (attention deficit and hyperactivity disorder), or comorbid disorders. Comorbid disorders were defined as meeting diagnostic criteria from two or more of the broad groups listed above. We did not identify those with Pervasive Developmental Disorder in ICD‐10 as clinical practice and the DAWBA changed between the 1999 and the 2004/2017 surveys; however, these children were not excluded from the ‘any disorder’ group as long as they met criteria for another disorder.

#### Sociodemographic and family characteristics

Baseline information related to child sex, age, and ethnicity (binary for main analysis, with an additional sensitivity analysis by categorical ethnic group), alongside parent report of housing tenancy (categorised into whether families owned or rented their homes), employment status (both parents working/one parent working/neither parent working), parental mental health (using the 12‐item General Health Questionnaire (GHQ12), dichotomised with a cut off score of 4; Goldberg & Williams, 1988), family type (single parent or not), family functioning (using the General Functioning Scale of the McMaster Family Assessment Device, in which problematic family functioning was indicated using a cut off of more than 2; Miller, Epstein, Bishop, & Keitner, 1985) and household income. Household income was asked using a showcard and included earnings from employment and receipt of benefits. Income was equivalised, adjusting for the number of residents, and split into quintiles and the bottom 20% within each of the three samples were compared with the other four. All variables were known correlates of mental health problems, or indicators of socioeconomic status. See Tables S1 and S2 for a comparison of baseline characteristics.

#### Difficulties and impact

Child difficulties were assessed across all surveys using the Strengths and Difficulties Questionnaire (SDQ) (Goodman, 1997). This comprises of 25 questions in five subscales; emotional symptoms, conduct problems, hyperactivity/inattention, peer relationship problems and prosocial behaviour. Each subscale is made up of 5 items, each rated on a three‐point scale. Total difficulty scores are generated by summing all items, other than those from the prosocial section, to form a score that ranges from 0 to 40. Higher scores indicate greater difficulties. Total difficulty scores were generated in all three samples using reports from the parent, the teacher and the child (aged 11–15 years only). The SDQ impact supplement comprises five items when rated by the parent or child, and three if rated by the teacher. The items assess overall distress and impairment and the degree to which these interfere with four aspects of life (friendship, classroom learning, home life and leisure activities). The five impact items are summed to generate an overall score that ranges from 0 to 10 for parent and child reports, and from 0 to 6 for teacher reports. A higher score indicates greater impact.

### Statistical analyses

The impact and difficulty levels of children having any disorder or no disorder were compared across surveys by testing mean differences in total difficulty scores and the impact scale from the SDQ. Regression models predicting SDQ scores by survey year were first conducted using parent and teacher reports across all individuals aged 5–15 years, and then repeated on children aged 5–10 years and 11–15 years. Weights were not used for these main analyses, as the weighting procedure for each survey to adjust for non‐response was slightly different. Application of available survey weights (using design weights adjusting for probability of selection and weighting for factors affecting non‐response, such as age and region) made no appreciable difference in a sensitivity analysis.

Logistic regressions were then used to examine the association between disorder status and demographic, socioeconomic or family correlates in each sample. Tests of interaction by survey year examined changes in the strength of these associations across the 18‐year period. A multivariable model with all predictors was used, with unadjusted univariable models also run with one correlate at a time. These models used participants with data for both the disorder status and individual correlate.

Additional analyses replicated the above logistic regressions after replacing the outcome of ‘any ICD‐10 disorder’ with either ‘any emotional disorder’, ‘any behavioural disorder’, ‘any hyperkinetic disorder’ or ‘any cross‐category comorbid disorders’. All analyses were conducted in Stata version 17 (StataCorp, 2021).

#### Sensitivity analyses

Comparisons of SDQ difficulty and impact scores were re‐run with entropy balanced weights. Entropy balancing is a multivariate re‐weighting method that calibrates unit weights such that two samples are balanced on a range of pre‐specified variables or covariates (Hainmueller & Xu, 2013). This was done to reduce the probability that any differences in difficulty or impact scores across samples are due to sample differences related to sociodemographic variables. We first compared difficulty and impact scores among the 1999 and 2004 surveys using entropy weights, and then repeated to compare 1999 to 2017, and 2004 to 2017. Variables used were baseline characteristics that were statistically different across the surveys being compared (Table S2). To further test the role of demographic changes over time, we repeated analyses after stratification by ethnicity. This was to account for sample differences in ethnicity across surveys.

## Results

### Descriptives

Information from parents was available for 99% of parents across the samples (1999 = 8,652, 2004 = 6,448 and 2017 = 6,216), while data collected from teachers were available for approximately 70% of those surveyed (1999 = 6,971, 2004 = 4,948 and 2017 = 3,338). For self‐reports, information was available for 88% of the sample of 11–15‐year‐olds (1999 = 3,540, 2004 = 2,609 and 2017 = 2,180). See Table S1 for more information about missing data. For children aged 5–15 years, small increases in prevalence were noted for those with any disorder between 1999 and 2004 (see Green, McGinnity, Meltzer, Ford, & Goodman, 2005 for full details), and further increases were observed in 2017 (Sadler et al., 2018).

### Comparison of difficulties of children by ICD‐10 disorder status across surveys

**Figure 1:**
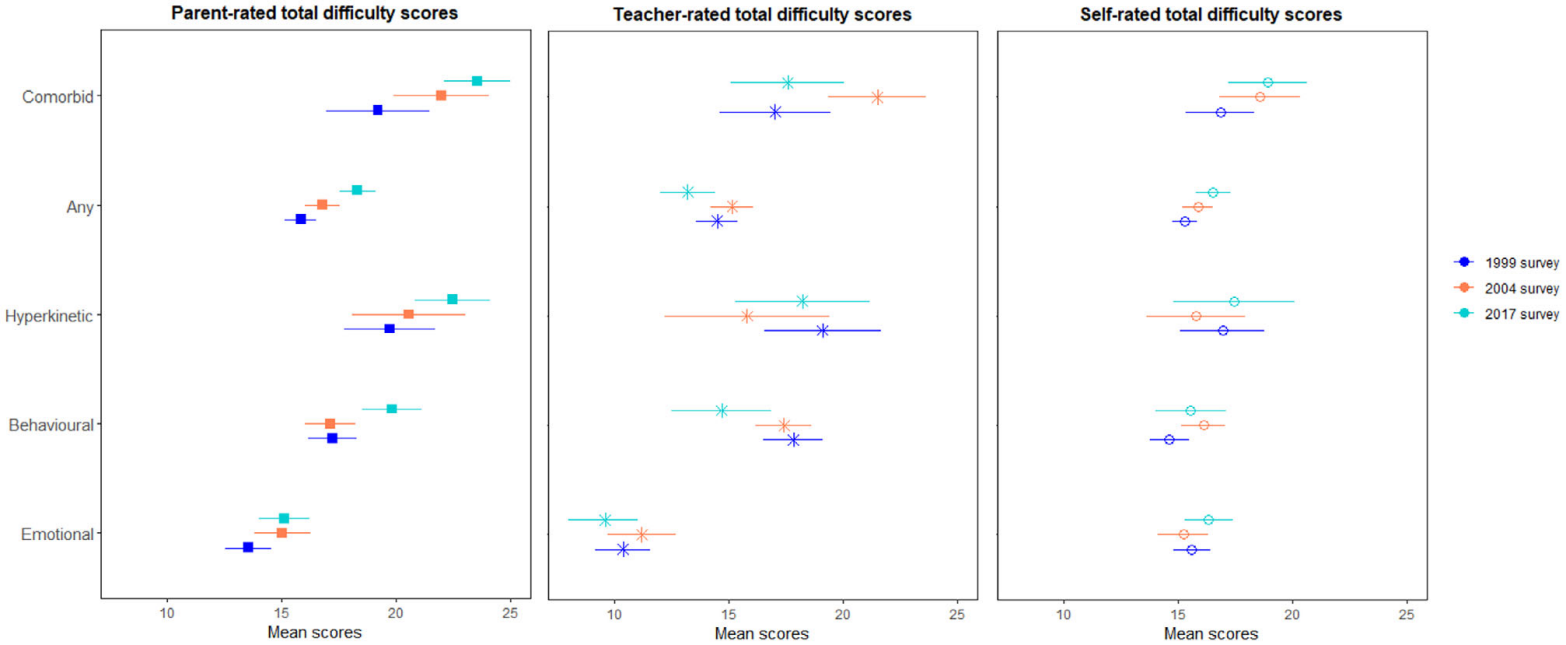
Cross survey comparison of parent‐, teacher‐ and self‐rated mean total difficulty scores (SDQ) among those aged 11–15. Comorbid disorders include those with at least two disorders, while any represents those with at least one

Parent‐rated SDQ total difficulty scores increased between 1999 and 2017 for children aged 5–15 years with any disorder (Figure S1), with consistent findings noted when using entropy balanced weights (see Table S4) among both 5–10 year olds (see Figure S2 and Table S5) and 11–15 year olds (see Figure 1 and Table S5). Increases for children aged 5–15 were also noted when specifically examining those with any emotional, behavioural or comorbid disorders (see Figures S1 and Tables S8–S11), but analyses stratified by age revealed increases were only consistent across the age range for those with a behavioural disorder (Table S9). For other disorders, increases were not consistently noted among those aged 5–10 years (Figure S2) or 11–15 year olds (Figure 1).

Teachers reported a reduction in total difficulty scores over time, with declines noted between 1999 and 2017, and between 2004 and 2017 for individuals aged 5–15 years with any disorder (see Figure S1). Note these differences were not significant when investigating children (Figure S2) and adolescents (Figure 1) separately. No differences were also noted in teacher‐reported difficulties for those with an emotional, hyperkinetic or comorbid disorders; however, these declined between 1999 and 2017, and between 2004 and 2017, for children and adolescents with a behavioural disorder (see Table S7 and Figure 1 and Figures S1 and S2).

For self‐reported difficulty scores, increased scores were noted between 1999 and 2017 for those with any disorder in 11–15 year olds (Figure 1), and between 1999 and 2004 for those with a behavioural disorder in both age groups (see Table S9).

For those without a disorder (Table 2), decreases in parent, teacher and self‐rated total difficulty scores were observed between 1999 and 2017.

Analyses stratified by ethnicity revealed that parent‐ and self‐reported increases in difficulty and impact scores for individuals with any disorder, are largely driven by trends noted among white participants (See Tables S6 and S7). Nevertheless, sample sizes for other ethnic groups were small, with wide confidence intervals around estimates; therefore, findings must be interpreted with caution.

### Comparison of impact of disorder among children by psychiatric disorder status across surveys

**Figure 2:**
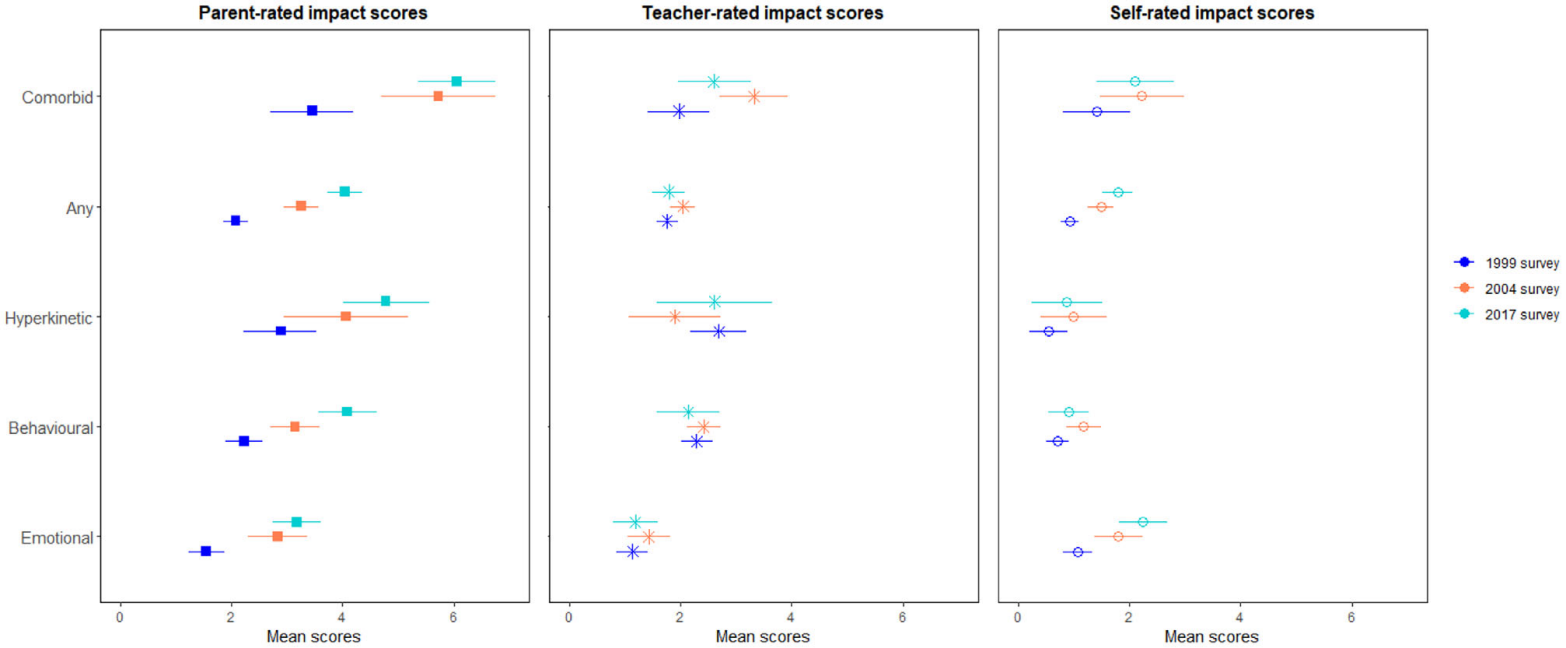
Cross survey comparison of parent‐, teacher‐ and self‐rated impact scores among those aged 11–15 years. Comorbid disorders include those with at least two disorders, while any represents those with at least one. Note impact scores for parent‐ and self‐reports range from 0 to 10, and for teacher reports from 0 to 6

SDQ impact scores also increased across the three samples when reported by parents, with increases found across the surveys for individuals aged 5–15 years with any disorder (see Table 1 and Figure S3). When examined by disorder category, parent ratings of impact increased between 1999 and 2017 and between 1999 and 2004 for those with an emotional, behavioural, hyperkinetic or comorbid disorder (see Tables S8–S11). Analyses stratified by age demonstrated that this was the case for both 5–10 year olds (Figure S4) and 11–15 year olds with an emotional, behavioural or comorbid disorder (see Figure 2).

**Table 1:** SDQ total difficulty and impact scores in 1999, 2004 and 2017, among 5–15 year olds with a DAWBA‐identified psychiatric disorder (England only)

	1999 (*n* = 769)	2004 (*n* = 577)	2017 (*n* = 619)	1999 vs. 2004	1999 vs. 2017	2004 vs. 2017
	Mean (95% CI)	Mean (95% CI)	Mean (95% CI)	Difference	Difference	Difference
*SDQ total difficulty score*						
Parent	16.38 (15.89, 16.87)	17.32 (16.75, 17.89)	18.53 (17.98, 19.09)	0.94 (0.19, 1.69)*	2.15 (1.42, 2.90)***	1.21 (0.42, 2.01)**
Teacher	14.41 (13.80, 15.03)	15.11 (14.39, 15.83)	13.16 (12.37, 13.95)	0.70 (−0.25, 1.64)	−1.25 (−2.26, −0.25)*	−1.95 (−3.02, −0.88)***
Self (11–15 only)	15.30 (14.77, 15.84)	15.87 (15.21, 16.52)	16.55 (15.79, 17.30)	0.56 (−0.27, 1.40)	1.25 (0.35, 2.14)**	0.68 (−0.31, 1.68)
*SDQ impact score*						
Parent	2.02 (1.86, 2.18)	3.28 (3.03, 3.52)	3.92 (3.69, 4.14)	1.25 (0.97, 1.54)***	1.90 (1.62, 2.16)***	0.64 (0.30, 0.98)***
Teacher	1.71 (1.58, 1.84)	2.02 (1.85, 2.19)	1.80 (1.60, 1.99)	0.31 (0.10, 0.52)**	0.09 (−0.14, 0.32)	−0.22 (−0.03, 0.48)
Self (11–15 only)	0.94 (0.78, 1.09)	1.49 (1.25, 1.72)	1.79 (1.51, 2.07)	0.55 (0.28, 0.82)***	0.86 (0.56, 1.15)***	0.30 (−0.06, 0.67)

Any disorder included any emotional, behavioural, hyperactive, or less common disorder identified using the DAWBA. Cohort interactions used to compare cohorts by SDQ score among those with a disorder. Impact scores when parent or self‐reported range from 0 to 10, while teacher impact scores range from 0 to 6. Self‐reports completed at 11–15 years only. ***p < .001; **p < .01; *p < .05.

No differences were found for teacher‐reported impact scores other than increases for 11–15 year olds with any disorder, or any comorbid disorder between 1999 and 2004 (see Figure 2). There were increases in self‐reported impact scores between 1999 and 2017, and between 1999 and 2004 for those with any disorder or any emotional disorder, and between 1999 and 2004 for those with a behavioural disorder (see also Figure 2).

For children without a disorder (Table 2), parent‐rated impact scores increased between 1999 and 2004, but were stable between 2004 and 2017. No changes were observed in teacher or self‐rated impact scores in this group.

**Table 2:** SDQ total difficulty and impact scores in 1999, 2004 and 2017 for whole samples (ages 5–15 years in England only) and those without a disorder

All participants	*N*	Mean (95% CI)	*N*	Mean (95% CI)	*N*	Mean (95% CI)	*p* Value	*p* Value	*p* Value
	1999 (*n* = 8,772)		2004 (*n* = 6,498)		2017 (*n* = 6,219)		1999 vs. 2004	1999 vs. 2017	2004 vs. 2017
*SDQ total difficulty score*									
Parent	8,652	8.46 (8.34, 8.59)	6,448	8.13 (7.98, 8.27)	6,216	8.06 (7.91, 8.22)	<.001	<.001	.54
Teacher	6,971	6.56 (6.42, 6.70)	4,948	6.58 (6.41, 6.75)	3,338	6.06 (5.86, 6.26)	.85	<.001	<.001
Self (11–15 only)	3,540	10.40 (10.23, 10.57)	2,609	10.17 (9.96, 10.37)	2,180	9.48 (9.25, 9.72)	.09	<.001	<.001
*SDQ impact score*									
Parent	8,654	0.39 (0.36, 0.41)	6,456	0.59 (0.55, 0.63)	6,219	0.67 (0.63, 0.71)	<.001	<.001	<.01
Teacher	6,923	0.41 (0.39, 0.43)	4,934	0.42 (0.40, 0.45)	3,339	0.46 (0.42, 0.49)	.40	<.05	.17
Self (11–15 only)	3,526	0.25 (0.22, 0.28)	2,595	0.33 (0.30, 0.37)	2,178	0.37 (0.32, 0.41)	<.001	<.001	.30

Those without a disorder	1999 (*n* = 8,000)		2004 (*n* = 5,921)		2017 (*n* = 5,600)		1999 vs. 2004	1999 vs. 2017	2004 vs. 2017
*SDQ total difficulty score*									
Parent	7,890	7.70 (7.59, 7.81)	5,876	7.23 (7.10, 7.36)	5,597	6.90 (6.78, 7.03)	<.001	<.001	<.001
Teacher	6,360	5.81 (5.68, 5.93)	4,511	5.76 (5.60, 5.91)	2,986	5.23 (5.04, 5.41)	.61	<.001	<.001
Self (11–15 only)	3,183	9.85 (9.68, 10.02)	2,349	9.54 (9.34, 9.74)	1,945	8.63 (8.41, 8.84)	<.05	<.001	<.001
*SDQ impact score*									
Parent	7,894	0.23 (0.21, 0.25)	5,883	0.33 (0.30, 0.36)	5,600	0.31 (0.28, 0.34)	<.001	<.001	.34
Teacher	6,311	0.28 (0.27, 0.30)	4,497	0.27 (0.25, 0.29)	2,987	0.30 (0.27, 0.33)	.33	.37	.10
Self (11–15 only)	3,170	0.17 (0.15, 0.19)	2,336	0.21 (0.18, 0.24)	1,944	0.19 (0.16, 0.22)	.06	.24	.55

### Comparison of demographic, socio‐economic and family characteristics of children with an ICD‐10 disorder across surveys

**Figure 3:**
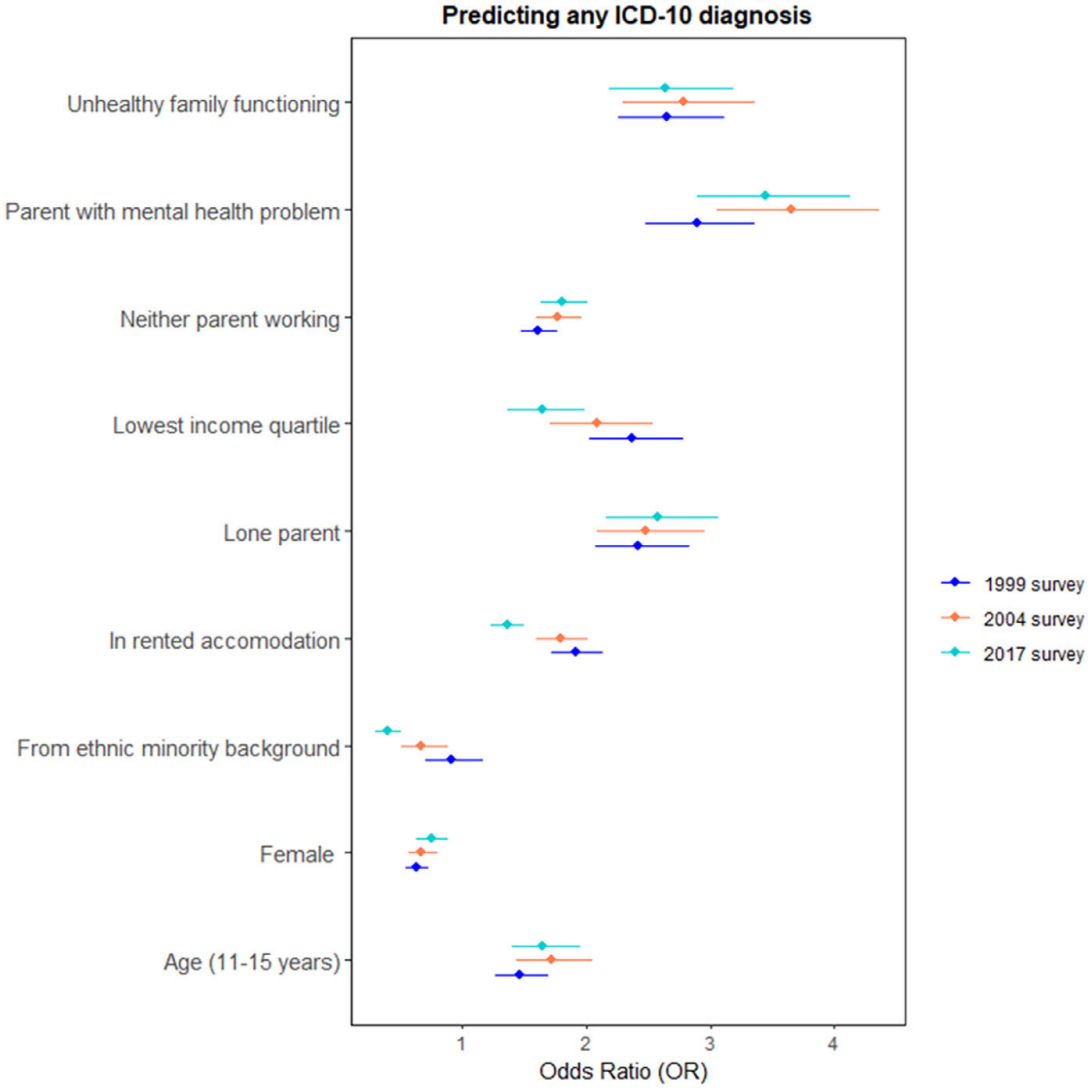
Cross survey comparison of odds of children having any ICD‐10 psychiatric disorder based on sociodemographic and family characteristics

Having a disorder was associated with a range of demographic, socioeconomic and family characteristics. Being male, older age, living in rented accommodation (compared with owner‐occupied), poor family functioning or parental mental health and being in the lowest income quintile were associated with an increased risk of disorder in all three surveys (see Table 3 and Figure 3).

**Table 3:** Multivariable model comparing sociodemographic and family characteristics of children with DAWBA‐identified psychiatric disorder

	1999 (*n* = 8,125)	2004 (*n* = 5,776)	2017 (*n* = 5,534)
	Estimate	Estimate	Estimate
Age (11–15 years %)^a^	1.39 (1.18, 1.64)***	1.80 (1.47, 2.19)***	1.57 (1.30, 1.90)***
Female (%)	0.62 (0.52, 0.72)***	0.67 (0.55, 0.82)***	0.73 (0.60, 0.88)***
From ethnic minority background (%)	0.89 (0.78, 1.01)	0.78 (0.67, 0.91)**	0.63 (0.54, 0.73)***
In rented accommodation (%)	1.52 (1.33, 1.73)***	1.32 (1.12, 1.55)***	1.14 (1.01, 1.30)*
Lowest income quintile (%)	1.02 (0.80, 1.30)	0.84 (0.62, 1.13)	0.79 (0.62, 1.02)
Neither parent working (%)	1.22 (1.08, 1.36)***	1.40 (1.21, 1.61)***	1.49 (1.30, 1.69)***
Lone parent (%)	1.53 (1.23, 1.89)***	1.57 (1.22, 2.02)***	1.99 (1.57, 2.52)***
Unhealthy family functioning (%)	1.92 (1.61, 2.29)***	1.94 (1.56, 2.42)***	1.95 (1.57, 2.42)***
Parent has mental health problem (%)	2.19 (1.85, 2.59)***	2.70 (2.20, 3.31)***	3.45 (2.89, 4.13)***

^a^ Reference category 5–10 year olds.

***p < .001; **p < .01; *p < .05.

No correlates became *more* strongly associated over time with an increased risk of a child having a disorder, except for white ethnicity. However, living in rented accommodation, or being in the lowest income quintile, had a weaker association with any disorder in 2017 compared to in 1999. Being from a minority ethnic background appeared to become more strongly associated with reduced odds of disorder over time and was consistent for different ethnic groups (Table S3).

Similar patterns of results were found when all variables were entered into one multivariable model (see Table S12), and also when examining sociodemographic correlates for each individual disorder group and comorbid disorders (see Tables S13–S17 and Figures S5–S8). The strength of association between older age and having an emotional disorder increased over time.

## Discussion

We analysed changes over time in the degree of difficulty and impact in children with a psychiatric disorder and changes in associations between risk of disorder and sociodemographic and family factors over an 18‐year period in England. We use data from three national child mental health probability sample surveys, which are unique in England in including psychiatric disorders assigned through clinical rating of standardised diagnostic assessments.

Our main finding was an increase in parent‐ and child‐reported difficulties, and an increase in the reported impact of those difficulties on functioning among children with a psychiatric disorder over time. Increases in awareness or propensity to report symptoms and functional impairment may explain our results. However, we did not find an increase in difficulties and impact to be universal across all age groups and disorders. In particular, we found that while parent‐reported impact increased between 1999 and 2017 for all disorders for individuals aged 11–15 years, self‐reported impact increased only for those with ‘any’ disorder, and any emotional disorder. These findings suggest that adolescents with an emotional disorder may be facing increased difficulties and would benefit the most from more targeted support. The findings also suggest that adolescents with a behavioural or hyperkinetic disorder may be less self‐aware of the impact of their disorder. Further research, however, is necessary to unpick why impact scores may vary across informants and disorder.

It is interesting to note that for individuals with a hyperkinetic disorder, no differences were found across time in parent‐reported difficulties, but there was an increase in parent‐rated impact. This suggests that while the difficulties faced by those with a hyperkinetic disorder have remained stable, the impact of having a disorder appears to have worsened over time. Understanding the underlying reasons for this will prove crucial to supporting those with a hyperkinetic disorder to improve their functional outcomes at school, home and in their daily lives.

The specificity in our findings for the different disorders mitigates against the argument that increases in disorder over time result largely from changes in awareness. Perhaps, more significantly, in those without disorder, there was a decrease in reported difficulties and only a minor increase in parent‐reported impact. We may suggest, therefore, that while changes in awareness may have contributed to our findings, they are unlikely to fully explain them. Indeed, work by McElroy et al. (2023) on measurement invariance of the SDQ over time suggested that parents in different cohorts interpreted the questions in a similar way, which may imply an increase in awareness.

One proposed alternative explanation for the increase in difficulty and impact scores among children with a disorder is that findings reflect differences over time in the demographic composition of the surveys. To account for this, we used entropy balancing to ensure surveys were weighted to account for demographic differences. Difficulty and impact scores following this were extremely similar. In addition, we accounted for one of the largest population shift across surveys. This was to determine whether larger samples of ethnic minority groups in more recent samples could account for differences in difficulty and impact over time. Analyses stratified by ethnicity revealed no clear evidence for trends in difficulty or impact scores over time for children with a disorder from non‐white ethnic backgrounds, with very wide confidence intervals around our estimates. The exception was a finding of increases in parent‐rated difficulties for Asian British and Asian groups. It is important to note, however, that sample sizes for non‐white ethnic groups with a disorder were extremely small, limiting our ability to interpret data on trends for children from other ethnic groups. Larger samples of children from ethnic minority groups are needed to examine trends. Without this, we potentially further disadvantage children from these groups by a lack of insight into changes in their mental health over time.

Overall, there were no changes in teacher‐rated total difficulty or impact scores for older children with a disorder over the three surveys. This is in line with prior research examining trends in teacher‐rated problems in the general population of children, which have tended to report stable or even decreasing teacher‐rated difficulties over time (Gutman, Joshi, Parsonage, & Schoon, 2018; Sellers et al., 2015). There are several factors that might explain such results, such as differences in where children express their difficulties (home vs. school) and to whom (parent vs. teacher). It is also possible that teachers' wider frame of reference has a bearing on their ratings of problems. For example, if they are becoming increasingly accustomed to witnessing emotional or behavioural symptoms in class, they may be less likely to rate these as impactful difficulties. Surprisingly low inter‐informant agreement on the SDQ and other similar measures is well established (Collishaw et al., 2019; Goodman et al., 2000; Murray, Speyer, Hall, Valdebenito, & Hughes, 2021).

We found some changes over time in the characteristics of children with a psychiatric disorder, and the correlates of disorder. While living in rented accommodation and living in a lower income household remained associated with an increased risk of disorder in all three surveys, they became less strongly associated over time. In contrast, being from an ethnic minority background became more strongly associated with a reduced risk of disorder between 1999 and 2017 (i.e. this emerged as a protective factor, while being of white ethnicity appeared associated with an increased risk).

These findings are challenging to explain. It is plausible that living in rented accommodation may have become a less strong marker of disadvantage or stigma for children between 1999 and 2017, as living in rented accommodation became much more common over the time scale of our study—rising from 6% in 1999 to 19% in 2017. Our findings on ethnicity may relate to a similar trend. The overall proportion of children in the sample from minority ethnic backgrounds rose in the period of our study, doubling from 10% in 1999 to 21% in 2017. It is possible that an increasingly multicultural society may improve outcomes for those from non‐white ethnic groups (Mirza & Warwick, 2022). This is a complex area, where intersections of ethnicity, religion, culture and other factors may also interact. Perhaps more importantly, ethnic inequalities in access to mental healthcare are well‐established in children and in adults, and may be widening, suggesting these groups remain under‐served by current policy and practice (Ahmad, McManus, Cooper, Hatch, & Das‐Munshi, 2022; Bansal et al., 2022).

Crucially, caution must be applied in interpreting our findings on socioeconomic correlates, particularly in the case of relative measures such as income quintiles, which are heavily influenced by changing policy and benefits structures for families in England (Stewart, Patrick, & Reeves, 2023; Wickham, Anwar, Barr, Law, & Taylor‐Robinson, 2016). There remains strong evidence of health (including mental health) disparities across a number of domains between children in low‐ and high‐income families in the UK, and other studies show no indication that these are narrowing over time (Collishaw, 2015; Marmot, Allen, Goldblatt, Herd, & Morrison, 2020; McElroy et al., 2023).

### Limitations

While our study has unique strengths, we also note several significant limitations. Firstly, we use a sample recruited from England, and our findings may not be generalisable to other geographical or cultural settings and contexts. Secondly, although all three surveys were designed to be representative, there were minor differences in sampling between the three surveys; 1999 and 2004 used the Child Benefit Register, and 2017 used the NHS Patient Register.

In common with many population surveys, response rates were lower in the more recent surveys (Ford et al., 2020), with 50% having complete data on all baseline characteristics and SDQ scores when based on teacher‐reports (see Table S1). This may affect survey comparison and the representativeness of the sample within these analyses. Note however, that there were small differences in missing data across survey waves for individual baseline characteristics, suggesting minimal bias due to select variables. There were some significant differences in the baseline characteristics of children across the three samples, as discussed above, although these appear to reflect broader population changes. We also note no differences were found between our findings after adjustment for sociodemographic differences across surveys through entropy balanced weights.

Thirdly, there were minor differences in the measures used across surveys, meaning that some sociodemographic measures were not available across samples. Fourthly, the broad scope of our study may obscure more nuanced trends by age, gender and disorder, exploration of which was beyond the remit of our planned analyses.

### Implications

One of the main and concerning implications of this study is that children with a disorder in the most recent survey experienced more severe difficulties and greater functional impact as rated by parents and children (although not by teachers), compared to children in earlier surveys. These findings are contrary to the view that observed increases in the prevalence of mental disorders largely reflect a societal shift of minor non‐consequential symptoms being ‘medicalised’ as disorder at lower thresholds of symptom difficulty or with less impairment in everyday functioning. It is plausible that with societal changes, a more pressurised school culture and changes in patterns of peer interactions, it is more difficult for children experiencing problems to successfully ‘function’ at the expected level. This may also contribute to increases in referrals and demand for child mental health services across various settings (Ball et al., 2023; Frith, 2017).

Given the sharp and sustained increase in probable disorder noted since the pandemic in England's national surveys (Newlove‐Delgado et al., 2022), this pre‐pandemic evidence of increased impact and poorer outcomes for those with disorder further underlines the urgency of maximising prevention and access to effective treatment. Furthermore, we emphasise that markers of socio‐economic disadvantage remained strongly associated with disorder. Evidence suggests that child health inequalities in England have widened in some domains over the past 10 years and are likely to have been further exacerbated by the pandemic and the subsequent cost of living crisis (Marmot et al., 2020). Research, prevention and intervention efforts must, therefore, continue to focus on monitoring and closing the gap and ensuring that policy and practice do not further widen inequalities. Research strategy should focus on coordinated, high quality and sustainable cross‐sectional and longitudinal research to be able to track, understand and address secular changes and their drivers.

## Conclusions

Our study shows that those with a disorder appear to experience increased difficulties and impacts in more recent generations. We note that these increased difficulties are specific to self‐ and parent‐reports and were not found when using teacher reports, and also that findings for children from ethnic minority backgrounds are less clear. Further work is required to understand the reasons behind these differences. These concerning findings underline the urgency for research and policy to identify and address the wider environmental, economic, educational and social influences that may be increasingly affecting children's ability to thrive and experience optimal outcomes.

**Key points**

- Research has previously described the profile of children with poor mental health, but little is known about whether this profile and the needs of children with a disorder have changed over time.
- Our study shows for the first time, that problems increased between 1999 and 2017 for children and adolescents with disorder.
- These concerning findings underline the urgency for research and policy to address the wider environmental, economic, educational and social influences that may be increasingly impacting a child's ability to thrive and experience optimal outcomes.

## Supporting information


**Table S1.** Survey sample sizes and missing data (England only).
**Table S2.** Survey sample baseline characteristics (England only).
**Table S3.** Comparison of ethnicity of children with any ICD‐10 disorder across surveys.
**Table S4.** Comparison of difficulties of children (5–10 years) and adolescents (11–15 years) with any ICD‐10 disorder.
**Table S5.** Comparison of difficulties of those aged 5–15 years with any ICD‐10 disorder, stratified by ethnicity.
**Table S6.** Comparison of impact of those aged 5–15 years with any ICD‐10 disorder, stratified by ethnicity.
**Table S7.** SDQ total difficulty scores in 1999, 2004 and 2017, among 5–15 year olds with a DAWBA‐identified psychiatric disorder (England only), controlling for entropy balanced weights.
**Table S8.** Comparison of difficulties of children (5–10 years) and adolescents (11–15 years) with any ICD‐10 emotional disorder.
**Table S9.** Comparison of difficulties of children (5–10 years) and adolescents (11–15 years) with any ICD‐10 behavioural disorder.
**Table S10.** Comparison of difficulties of children (5–10 years) and adolescents (11–15 years) with any ICD‐10 hyperkinetic disorder.
**Table S11.** Comparison of difficulties of children (5–10 years) and adolescents (11–15 years) with any ICD‐10 comorbid disorder.
**Table 12.** Comparison of sociodemographic and family characteristics of children with DAWBA‐identified psychiatric disorder (univariable models).
**Table S13.** Comparison of characteristics of children with any emotional disorder (all ages).
**Table S14.** Comparison of characteristics of children with any emotional disorder (11–15 years).
**Table S15.** Comparison of characteristics of children with any behavioural disorder (all ages).
**Table S16.** Comparison of characteristics of children with any hyperkinetic disorder (all ages).
**Table S17.** Comparison of characteristics of children with any cross‐comorbid disorder (all ages).
**Figure S1.** Cross survey comparison of parent‐ and teacher‐rated mean total difficulty scores (SDQ) among those aged 5–15 years.
**Figure S2.** Cross survey comparison of parent‐ and teacher‐rated mean total difficulty scores (SDQ) among those aged 5–10 years.
**Figure S3.** Cross survey comparison of parent and teacher‐rated impact scores among those aged 5–15 years. Note impact scores for parent reports range from 0–10, and for teacher reports from 0–6.
**Figure S4.** Cross survey comparison of parent and teacher‐rated impact scores among those aged 5–10 years. Note impact scores for parent reports range from 0–10, and for teacher reports from 0–6.
**Figure S5.** Odds of children having any emotional disorder based on sociodemographic and family characteristics.
**Figure S6.** Odds of children having any behavioural disorder based on sociodemographic and family characteristics.
**Figure S7.** Odds of children having any hyperkinetic disorder based on sociodemographic and family characteristics.
**Figure S8.** Odds of children having any comorbid disorder based on sociodemographic and family characteristics.
