# Supplementary material for: Characteristics of children with a psychiatric disorder in 1999, 2004 and 2017: an analysis of the national child mental health surveys of England
Source: J Child Psychol Psychiatry. 2024 Jul 24;66(2):167–77. doi: 10.1111/jcpp.14040 (PMC11754707; doi:10.1111/jcpp.14040)

**Characteristics of children with a psychiatric disorder in 1999, 2004 and 2017: An analysis of the national child mental health surveys of England**

**Supporting Information**

**Tables**

- **Table S1**: Survey sample sizes and missing data (England only)
- **Table S2**: Survey sample baseline characteristics (England only)
- **Table S3:** Comparison of ethnicity of children with any ICD-10 disorder across surveys
- **Table S4:** Comparison of difficulties of children (5-10 years) and adolescents (11-15 years) with any ICD-10 disorder
- **Table S5:** Comparison of difficulties of those aged 5-15 years with any ICD-10 disorder, stratified by ethnicity
- **Table S6:** Comparison of impact of those aged 5-15 years with any ICD-10 disorder, stratified by ethnicity
- **Table S7:** SDQ total difficulty scores in 1999, 2004 and 2017, among 5-15 year olds with a DAWBA-identified psychiatric disorder (England only), controlling for entropy balanced weights
- **Table S8:** Comparison of difficulties of children (5-10 years) and adolescents (11-15 years) with any ICD-10 emotional disorder
- **Table S9:** Comparison of difficulties of children (5-10 years) and adolescents (11-15 years) with any ICD-10 behavioural disorder
- **Table S10:** Comparison of difficulties of children (5-10 years) and adolescents (11-15 years) with any ICD-10 hyperkinetic disorder
- **Table S11:** Comparison of difficulties of children (5-10 years) and adolescents (11-15 years) with any ICD-10 comorbid disorder
- **Table 12:** Comparison of sociodemographic and family characteristics of children with DAWBA-identified psychiatric disorder (univariable models)
- **Table S13:** Comparison of characteristics of children with any emotional disorder (all ages)
- **Table S14**: Comparison of characteristics of children with any emotional disorder (11-15 years)
- **Table S15:** Comparison of characteristics of children with any behavioural disorder (all ages)
- **Table S16:** Comparison of characteristics of children with any hyperkinetic disorder (all ages)
- **Table S17**: Comparison of characteristics of children with any cross-comorbid disorder (all ages)

**Figures**

- **Figure S1**: Cross survey comparison of parent- and teacher-rated mean total difficulty scores (SDQ) among those aged 5-15 years
- **Figure S2**: Cross survey comparison of parent- and teacher-rated mean total difficulty scores (SDQ) among those aged 5-10 years
- **Figure S3**: Cross survey comparison of parent and teacher-rated impact scores among those aged 5-15 years. Note impact scores for parent reports range from 0-10, and for teacher reports from 0-6.
- **Figure S4**: Cross survey comparison of parent and teacher-rated impact scores among those aged 5-10 years. Note impact scores for parent reports range from 0-10, and for teacher reports from 0-6.
- **Figure S5**: Odds of children having any emotional disorder based on sociodemographic and family characteristics
- **Figure S6**: Odds of children having any behavioural disorder based on sociodemographic and family characteristics
- **Figure S7**: Odds of children having any hyperkinetic disorder based on sociodemographic and family characteristics
- **Figure S8**: Odds of children having any comorbid disorder based on sociodemographic and family characteristics

| **Table S1**: **Survey sample sizes and missing data (England only)** | | | |
| --- | --- | --- | --- |
|  | **1999** | **2004** | **2017** |
| Total sample at baseline (5-15 years)  *% Aged 5-10 years*  *% Aged 11-15 years* | 8,772  5,014 (57.2%)  3,758 (42.8%) | 6,498  3,453 (53.1%)  3,045 (46.9%) | 6,219  3,597 (57.4%)  2,622 (42.6%) |
| % Aged 5-15 years with complete SDQ data – parent report | 8,652 (98.6%) | 6,448 (99.2%) | 6,216 (99.9%) |
| % Aged 5-15 years with complete SDQ data – teacher report | 6,971 (79.5%) | 4,948 (76.1%) | 3,338 (53.7%) |
| % Aged 11-15 years with complete SDQ data – self-report | 3,540 (94.1%) | 2,609 (85.7%) | 2,180 (83.1%) |
| % Aged 5-15 years with complete data on disorder status and baseline characteristics  *% Aged 5-15 years with complete data on disorders status and ethnicity*  *% Aged 5-15 years with complete data on disorders status and housing*  *% Aged 5-15 years with complete data on disorders status and parental work*  *% Aged 5-15 years with complete data on disorders status and lone parent*  *% Aged 5-15 years with complete data on disorders status and income*  *% Aged 5-15 years with complete data on disorders status and family functioning*  *% Aged 5-15 years with complete data on disorders status and parent mental health* | 8,125 (92.6%)  8,765 (99.9%)  8,766 (99.9%)  8,664 (98.8%)  8,772 (100%)  8,205 (93.5%)  8,580 (97.8%)  8,614 (98.2%) | 5,776 (88.9%)  6,494 (99.9%)  6,495 (99.9%)  6,350 (97.7%)  6,498 (100%)  5,827 (89.7%)  6,264 (96.4%)  6,292 (96.8%) | 5,534 (90.0%)  6,217 (99.9%)  6,210 (99.9%)  6,193 (99.6%)  6,219 (100%)  5,590 (89.9%)  6,099 (98.1%)  6,122 (98.4%) |
| % Aged 5-15 years with complete data on baseline characteristics and SDQ (parent) | 8,121 (92.6%) | 5,774 (88.9%) | 5,532 (90.0%) |
| % Aged 5-15 years with complete data on all characteristics and SDQ (teacher) | 6,490 (74.0%) | 4,451 (68.5%) | 3,103 (50.0%) |
| % Aged 11-15 years with complete data on all characteristics and SDQ (self) | 3,264 (86.9%) | 2,323 (76.3%) | 1,958 (74.7%) |
| Note: Characteristics include age, sex, ethnicity, housing, parent work status, income, lone parent status, family functioning, and parental mental health | | | |

| **Table S2**: **Survey sample characteristics (England only)** | | | | | | |
| --- | --- | --- | --- | --- | --- | --- |
|  | 1999  (n=8,772) | 2004  (n=6,498) | 2017  (n=6,219) | 1999 v 2004 | 1999 v 2017 | 2004 v 2017 |
| **Baseline characteristics** | | | | | | |
| Sex (female), % | 50.22 (49.16, 51.26) | 48.15 (46.93, 49.38) | 49.72 (48.47, 50.97) | <0.05 | 0.55 | 0.08 |
| Age, mean | 9.84 (9.78, 9.91) | 10.09 (10.02, 10.17) | 9.76 (9.68, 9.84) | <0.001 | 0.13 | <0.001 |
| N and % aged 5-10 | 5,014 (57.2%) | 3,453 (49.20%) | 3,597 (57.8%) | <0.001 | 0.46 | <0.001 |
| <N and % aged 11-15 | 3,758 (42.84%) | 4,052 (50.8%) | 2,622 (42.2%) | <0.001 | 0.44 | <0.001 |
| Ethnicity (minority), % | 9.94 (9.31, 10.57) | 14.45 (13.60, 15.31) | 20.94 (19.92, 21.95) | <0.001 | <0.001 | <0.001 |
| Housing (in rented accommodation), % | 6.30 (5.80, 6.83) | 7.48 (6.85, 8.15) | 18.78 (17.81, 19.77) | <0.001 | <0.001 | <0.001 |
| Neither parent working, % | 13.41 (12.70, 14.15) | 15.91 (15.01, 16.83) | 11.22 (10.45, 12.03) | <0.001 | <0.001 | <0.001 |
| Lowest income quintile (%) | 23.07 (22.16, 23.98) | 20.30 (19.27, 21.33) | 23.60 (22.48, 24.71) | <0.001 | 0.47 | <0.001 |
| Lone parent, % | 22.30 (21.43, 23.18) | 24.30 (23.26, 25.36) | 21.40 (20.39, 22.44) | <0.01 | 0.19 | <0.001 |
| **Family functioning** | | | | | | |
| Unhealthy family functioning, % | 18.76 (17.94, 19.61) | 17.31 (16.38, 18.27) | 16.31 (15.39, 17.27) | <0.05 | <0.001 | 0.14 |
| Parent has mental health problem, % | 25.09 (24.17, 26.02) | 22.47 (21.45, 23.52) | 19.03 (18.05, 20.04) | <0.001 | <0.001 | <0.001 |

| **Table S3**: Comparison of ethnicity of children with DAWBA-identified psychiatric disorder | | | | | | | | | | | | |
| --- | --- | --- | --- | --- | --- | --- | --- | --- | --- | --- | --- | --- |
|  | 1999 (n=8,772) | | | 2004 (n=6,498) | | | 2017 (n=6,219) | | | Survey interaction | | |
|  | With disorder  (n=769)  (%) | Without disorder  (n=8,003)  (%) | OR  (95% CI) | With disorder  (n=577)  (%) | Without disorder  (n=5,921)  (%) | OR  (95% CI) | With disorder  (n=619)  (%) | Without disorder  (n=5,600)  (%) | OR  (95% CI) | 1999 v 2004  (OR, 95% CI) | 1999 v 2017  (OR , 95% CI) | 2004 v 2017  (OR, 95% CI) |
| White | 90.9 | 90.1 | Reference | 89.6 | 85.2 | Reference | 90.0 | 77.9 | Reference |  |  |  |
| Black British / Caribbean / African | 2.5 | 1.9 | 1.28 (0.79, 2.08) | 1.6 | 2.6 | 0.56 (0.29, 1.11) | 1.8 | 4.5 | 0.34 (0.18, 0.62)*** | 0.44 (0.19, 1.01) | 0.26 (0.12, 0.58)*** | 0.60 (0.24, 1.50) |
| Asian British / Asian | 2.7 | 4.6 | 0.60 (0.38, 0.93)* | 4.2 | 7.3 | 0.55 (0.36, 0.83)** | 2.8 | 11.0 | 0.22 (0.13, 0.35)*** | 0.92 (0.50, 1.69) | 0.36 (0.19, 0.70)*** | 0.40 (0.21, 0.76)** |
| Mixed or other | 3.9 | 3.5 | 1.11 (0.76, 1.64) | 4.7 | 4.9 | 0.91 (0.61, 1.36) | 5.5 | 6.6 | 0.72 (0.50, 1.04) | 0.82 (0.47, 1.43) | 0.65 (0.38, 1.10) | 0.80 (0.46, 1.37) |
| Note: Being of white ethnicity was used as the baseline comparison group. | | | | | | | | | | | | |

| **Table S4:** SDQ total difficulty scores in 1999, 2004 and 2017, among 5-15 year olds with a DAWBA-identified psychiatric disorder (England only), controlling for entropy balanced weights | | | |
| --- | --- | --- | --- |
|  | 1999 (n=769) | 2004 (n=577) | 2017 (n=619) |
| **SDQ total difficulty score** | Mean (95% CI) | Mean (95% CI) | Mean (95% CI) |
| **Parent** | | | |
| Unadjusted | 16.38 (15.89, 16.87) | 17.32 (16.75, 17.89) | 18.53 (17.98, 19.09) |
| Weighted using 1999 survey | - | 17.23 (16.61, 17.85) | 18.29 (17.69, 18.88) |
| Weighted using 2004 survey | 16.42 (15.91, 16.92) | - | 18.31 (17.72, 18.91) |
| Weighted using 2017 survey | 16.48 (15.98, 16.98) | 17.21 (16.57, 17.84) | - |
| **Teacher** | | | |
| Unadjusted | 14.41 (13.80, 15.03) | 15.11 (14.39, 15.83) | 13.16 (12.37, 13.95) |
| Weighted using 1999 survey | - | 15.07 (14.28, 15.85) | 13.21 (12.40, 14.01) |
| Weighted using 2004 survey | 14.38 (13.75, 15.00) | - | 13.27 (12.47, 14.07) |
| Weighted using 2017 survey | 14.57 (13.95, 15.19) | 15.02 (14.26, 15.78) | - |
| **Self (11-15 only)** | | | |
| Unadjusted | 15.30 (14.77, 15.84) | 15.87 (15.21, 16.52) | 16.55 (15.79, 17.30) |
| Weighted using 1999 survey | - | 15.84 (15.13, 16.55) | 16.56 (15.77, 17.35) |
| Weighted using 2004 survey | 15.25 (14.69, 15.79) | - | 16.61 (15.81, 17.40) |
| Weighted using 2017 survey | 15.31 (14.77, 15.85) | 15.64 (14.93, 16.34) | - |
| **SDQ impact score** |  |  |  |
| **Parent** | | | |
| Unadjusted | 2.02 (1.86, 2.18) | 3.28 (3.03, 3.52) | 3.92 (3.69, 4.14) |
| Weighted using 1999 survey | - | 3.22 (2.95, 3.48) | 3.83 (3.59, 4.07) |
| Weighted using 2004 survey | 2.05 (1.88, 2.21) | - | 3.87 (3.63, 4.11) |
| Weighted using 2017 survey | 2.01 (1.85, 2.17) | 3.23 (2.96, 3.50) | - |
| **Teacher** | | | |
| Unadjusted | 1.71 (1.58, 1.84) | 2.02 (1.85, 2.19) | 1.80 (1.60, 1.99) |
| Weighted using 1999 survey | - | 2.00 (1.82, 2.18) | 1.82 (1.61, 2.03) |
| Weighted using 2004 survey | 1.72 (1.58, 1.85) | - | 1.82 (1.62, 2.03) |
| Weighted using 2017 survey | 1.72 (1.58, 1.85) | 2.01 (1.84, 2.19) | - |
| **Self (11-15 only)** | | | |
| Unadjusted | 0.94 (0.78, 1.09) | 1.49 (1.25, 1.72) | 1.79 (1.51, 2.07) |
| Weighted using 1999 survey | - | 1.45 (1.20, 1.69) | 1.82 (1.52, 2.12) |
| Weighted using 2004 survey | 0.95 (0.78, 1.12) | - | 1.86 (1.56, 2.16) |
| Weighted using 2017 survey | 0.92 (0.76, 1.09) | 1.45 (1.21, 1.70) |  |
| Note: †Any disorder included any emotional, behavioural, hyperactive, or less common disorder identified using the DAWBA. Impact scores when parent or self-reported range from 0 to 10, while teacher impact scores range from 0 to 6. Self-reports completed at 11-15 years only. | | | |

| **Table S5:** Comparison of difficulties of children (5-10 years) and adolescents (11-15 years) with any ICD-10 disorder | | | | | | |
| --- | --- | --- | --- | --- | --- | --- |
|  | Cohort of children with ICD-10 disorder | | | Survey Comparisons | | |
|  | 1999 | 2004 | 2017 | 1999 v 2004 | 1999 v 2017 | 2004 v 2017 |
| **SDQ total difficulty score** | Mean (95% CI) | Mean (95% CI) | Mean (95% CI) |  |  |  |
| Parent (5-10) | 16.93 (16.23, 17.62) | 18.06 (17.19, 18.93) | 18.77 (17.99, 19.55) | 1.13 (0.19, 2.24)* | 1.84 (0.80, 2.89)*** | 0.71 (-0.45, 1.88) |
| Parent (11-15) | 15.86 (15.16, 16.55) | 16.81 (16.05, 17.56) | 18.33 (17.54, 19.12) | 0.95 (-0.73, 1.97) | 2.47 (1.43, 3.52)*** | 1.52 (0.43, 2.61)** |
| Teacher (5-10) | 14.34 (13.49, 15.19) | 15.06 (13.95, 16.18) | 13.12 (12.06, 14.17) | 0.72 (-0.68, 2.12) | -1.22 (-2.59, 0.149) | -1.95 (-3.48, - 0.42)*** |
| Teacher (11-15) | 14.49 (13.60, 15.38) | 15.14 (14.19, 16.08) | 13.20 (12.00, 14.41) | 0.65 (-0.65, 1.94) | -1.29 (-2.77, 0.200) | -1.94 (-3.45, -0.42)* |
| Self (11-15 only) | 15.30 (14.77, 15.84) | 15.87 (15.21, 16.52) | 16.55 (15.79, 17.30) | 0.56 (0.27, 1.40) | 1.25 (0.35, 2.14)** | 0.68 (0.31, 1.68) |
| **SDQ impact score** |  |  |  |  |  |  |
| Parent (5-10) | 1.97 (1.73, 2.20) | 3.29 (2.90, 3.69) | 3.77 (3.44, 4.10) | 1.32 (0.90, 1.75)*** | 1.80 (1.41, 2.19)*** | 0.48 (-0.03, 0.99) |
| Parent (11-15) | 2.08 (1.86, 2.30) | 3.26 (2.94, 3.58) | 4.05 (3.73, 4.36) | 1.19 (0.81, 1.57)*** | 1.97 (1.59, 2.34)*** | 0.78 (0.33, 1.23)*** |
| Teacher (5-10) | 1.66 (1.48, 1.85) | 1.98 (1.74, 2.23) | 1.80 (1.52, 2.07) | 0.32 (0.02, 0.63)* | 0.13 (-0.18, 0.45) | -0.18 (-0.55, 0.18) |
| Teacher (11-15) | 1.76 (1.57, 1.95) | 2.05 (1.82, 2.27) | 1.79 (1.50, 2.09) | 0.29 (-0.03, 0.58) | 0.03 (-0.30, 0.37) | -0.25 (-0.62, 0.11) |
| Self (11-15 only) | 0.94 (0.78, 1.09) | 1.49 (1.25, 1.72) | 1.79 (1.51, 2.07) | 0.55 (0.28, 0.82)*** | 0.86 (0.56, 1.15)*** | 0.30 (-0.06, 0.67) |
| Note: Impact scores when parent or self-reported range from 0 to 10, while teacher impact scores range from 0 to 6. Self-reports completed at 11-15 years only. Observations for children with any disorder were n=374 in 1999, n=236 in 2004, and n=289 in 2017. For adolescents, observations were n=395 in 1999, n=341 in 2004, n=330 in 2017. Note these samples vary depending on informant. | | | | | | |

| **Table S6:** Comparison of difficulties of those aged 5-15 years with any ICD-10 disorder, stratified by ethnicity | | | | | | | | | |
| --- | --- | --- | --- | --- | --- | --- | --- | --- | --- |
|  | Cohort of children with ICD-10 disorder | | | | | | Survey Comparisons | | |
|  | 1999 | | 2004 | | 2017 | | 1999 v 2004 | 1999 v 2017 | 2004 v 2017 |
| **SDQ total difficulty score** | N | Mean (95% CI) | N | Mean (95% CI) | N | Mean (95% CI) |  |  |  |
| **Parent-rated, by ethnicity (5-15 years)** | | | | | | | | | |
| White | 670 | 16.55  (16.03, 17.07) | 515 | 17.51  (16.91, 18.12) | 560 | 18.70  (18.11, 19.28) | 0.96 (0.16, 1.76)* | 2.15 (1.37, 2.93)*** | 1.19 (0.34, 2.02)** |
| Black British / Caribbean / African | 20 | 15.26  (12.01, 18.51) | <10 | 16.33  (12.28, 20.38) | 10 | 13.18  (8.86, 17.50) | 1.07 (-4.14, 6.28) | -2.08 (-6.96, 2.80) | -3.15 (-8.76, 2.46) |
| Asian British / Asian | 20 | 13.47  (10.23, 16.72) | 20 | 15.48  (12.91, 18.04) | 20 | 18.41  (15.06, 21.76) | 2.01 (-1.98, 5.99) | 4.94 (0.74, 9.14)* | 2.93 (-1.06, 6.93) |
| Mixed or other | 30 | 15.25  (12.75, 17.75) | 30 | 15.44  (12.67, 18.21) | 35 | 17.71  (15.34, 20.07) | 0.19 (-3.42, 3.81) | 2.46 (-0.97, 5.88) | 2.26 (-1.29, 5.81) |
| **Teacher-rated, by ethnicity (5-15 years)** | | | | | | | | | |
| White | 560 | 14.46  (13.82, 15.10) | 390 | 15.19  (14.42, 15.95) | 320 | 13.44  (12.61, 14.28) | 0.73 (-0.27, 1.72) | -1.02 (-2.07, 0.03) | -1.74 (-2.88, -0.61)** |
| Black British / Caribbean / African | 15 | 14.21  (8.82, 19.61) | <10 | 12.33  (7.01, 17.66) | <5 | s | -1.88 (-9.13, 5.37) | -7.81 (-16.65, 1.03) | -5.93 (-14.24, 2.38) |
| Asian British / Asian | 20 | 12.12  (8.57, 15.67) | 20 | 13.16  (9.44, 16.87) | <10 | 11.71  (6.17, 17.26) | 1.04 (-3.79, 5.87) | -0.41 (-6.90, 6.09) | -1.45 (-8.12, 5.24) |
| Mixed or other | 20 | 15.47  (11.66, 19.28) | 20 | 16.55  (13.26, 19.83) | 20 | 10.68  (7.55, 13.82) | 1.07 (-3.51, 5.65) | -4.79 (-9.54, -0.04)* | -5.86 (-10.30, -1.42)* |
| **Self-rated, by ethnicity**  **(11-15 years)** | | | | | | | | | |
| White | 325 | 15.46  (14.90, 16.01) | 230 | 15.98  (15.27, 16.70) | 210 | 16.63  (15.84, 17.43) | 0.52 (-0.39, 1.44) | 1.17 (0.23, 2.12)* | 0.65 (-0.41, 1.71) |
| Black British / Caribbean / African | <10 | 12.38  (8.58, 16.17) | <5 | s | <10 | 16.17  (5.60, 26.73) | 3.12 (-5.74, 11.99) | 3.79 (-4.03, 11.61) | 0.67 (-11.65, 12.98) |
| Asian British / Asian | 10 | 14.42  (9.70, 19.14) | <10 | 16.00  (12.42, 19.58) | <10 | 14.83  (10.56, 19.10) | 1.58 (-4.29, 7.46) | 0.42 (-5.76, 6.59) | -1.17 (-6.02, 3.69) |
| Mixed or other | 15 | 14.14  (12.02, 16.27) | 20 | 14.29  (12.30, 16.29) | 15 | 16.21  (13.12, 19.31) | 0.15 (-3.01, 3.31) | 2.07 (-1.24, 5.38) | 1.92 (-1.48, 5.32) |
| Note: Observations for children with any disorder were n=374 in 1999, n=236 in 2004, and n=289 in 2017. For adolescents, observations were n=395 in 1999, n=341 in 2004, n=330 in 2017. Note these samples vary depending on informant. Statistical Disclosure Control guidelines from NHS England have been applied to these data. | | | | | | | | | |

| **Table S7:** Comparison of impact of those aged 5-15 years with any ICD-10 disorder, stratified by ethnicity | | | | | | | | | |
| --- | --- | --- | --- | --- | --- | --- | --- | --- | --- |
|  | Cohort of children with ICD-10 disorder | | | | | | Survey Comparisons | | |
|  | 1999 | | 2004 | | 2017 | | 1999 v 2004 | 1999 v 2017 | 2004 v 2017 |
| **SDQ impact score** | N | Mean (95% CI) | N | Mean (95% CI) | N | Mean (95% CI) |  |  |  |
| **Parent-rated, by ethnicity (5-15 years)** | | | | | | | | | |
| White | 695 | 2.06  (1.89, 2.23) | 515 | 3.33  (3.07, 3.59) | 555 | 3.97  (3.73, 4.21) | 1,27 (0.97, 1.58)*** | 1.91 (1.60, 2.21)*** | 0.63 (0.28, 0.99)** |
| Black British / Caribbean / African | 20 | 1.79  (0.90, 2.68) | <10 | 2.44  (0.1, 4.9) | 10 | 3.0  (0.56, 5.4) | 0.65 (-1.61, 2.92) | 1.21 (-0.1, 3.33) | 0.56 (-2.69, 3.80) |
| Asian British / Asian | 20 | 1.0  (0.13, 1.87) | 20 | 1.71  (0.53, 2.90) | 20 | 4.47  (2.81, 6.13) | 0.71 (-0.93, 2.35) | 3.47 (1.74, 5.20) | 2.76 (0.84, 4.68) |
| Mixed or other | 30 | 2.04  (1.07, 3.01) | 30 | 3.67  (2.37, 4.97) | 35 | 3.11  (2.31, 3.93) | 1.63  (0.18, 3.08) | 1.08  (-0.29, 2.45) | -0.55  (-1.99, 0.89) |
| **Teacher-rated, by ethnicity (5-15 years)** | | | | | | | | | |
| White | 560 | 1.71  (1.58, 1.85) | 370 | 2.04  (1.87, 2.23) | 320 | 1.86  (1.65, 2.08) | 0.33 (0.11, 0.57)*** | 0.15 (-0.93, 0.39) | -0.19 (-0.46, 0.87) |
| Black British / Caribbean / African | 15 | 1.79  (0.90, 2.66) | <10 | 1.33  ( 0.52, 2.72) | <10 | 1.4  (-1.02, 3.82) | -0.45 (-1.94, 1.04) | -0.39 (-2.20, 1.43) | 0.07 (-2.12, 2.32) |
| Asian British / Asian | 15 | 1.53  (0.92, 2.14) | 20 | 1.56  (0.79, 2.32) | <10 | 1.0  (0.75, 1.92) | 0.026  (-.08, 0.93) | -0.53  (-1.73, 0.68) | -0.56  (-1.86, 0.75) |
| Mixed or other | 20 | 1.79  (0.87, 2.71) | 20 | 2.19  (1.39, 2.98) | 20 | 1.05  (0.42, 1.86) | 0.39  (-0.73, 1.51) | -0.74  (-1.90, 0.43) | -1.13 **  (-2.23, -0.27) |
| **Self-rated, by ethnicity**  **(11-15 years)** | | | | | | | | | |
| White | 320 | 0.94  (0.77, 1.11) | 230 | 1.47  (1.22, 1.71) | 210 | 1.76  (1.47, 2.06) | 0.53  (0.22, 0.84)*** | 0.83  (0.50, 1.15)*** | 0.30  (-0.86, 0.68) |
| Black British / Caribbean / African | <10 | 0.88  (0.47, 1.70) | <5 | s | <10 | 1.83  (-0.51, 4.17) | 0.88  (-1.16, 2.91) | 0.96  (-0.84, 2.75) | 0.08  (-2.78, 2.95) |
| Asian British / Asian | 10 | 1.08  (-0.16, 2.18) | <10 | 2  (0.31, 3.69) | <10 | 2.33  (0.62, 4.05) | 0.92  (-0.79, 2.63) | 1.25  (-0.55, 3.05) | 0.33  (-1.80, 2.47) |
| Mixed or other | 15 | 0.79  (0.10, 1.48) | 20 | 1.47  (0.33, 2.61) | 15 | 1.92  (0.52, 3.33) | 0.68  (-0.80, 2.17) | 1.14  (-0.41, 2.69) | 0.46  (-1.25, 2.17) |
| Note: Observations for children with any disorder were n=374 in 1999, n=236 in 2004, and n=289 in 2017. For adolescents, observations were n=395 in 1999, n=341 in 2004, n=330 in 2017. Note these samples vary depending on informant. Statistical Disclosure Control guidelines from NHS England have been applied to these data. | | | | | | | | | |

| **Table S8:** Comparison of difficulties of children (5-10 years) and adolescents (11-15 years) with any ICD-10 emotional disorder | | | | | | |
| --- | --- | --- | --- | --- | --- | --- |
|  | Cohort of children with ICD-10 emotional disorder | | | Survey Comparisons | | |
|  | 1999 | 2004 | 2017 | 1999 v 2004 | 1999 v 2017 | 2004 v 2017 |
| **SDQ total difficulty score (mean, 95% CI)** |  |  |  |  |  |  |
| Parent (all ages) | 13.74 (13.02, 14.45) | 15.53 (14.58, 16.47) | 15.28 (14.43, 16.13) | 1.80 (0.61, 2.97)** | 1.54 (0.44, 2.65)** | -0.25 (-1.53, 1.04) |
| Parent (5-10) | 13.95 (12.92, 14.97) | 16.56 (15.06, 18.05) | 15.52 (14.20, 16.85) | 2.61 (0.76, 4.46)** | 1.58 (-0.07, 3.22) | -1.04 (-3.07, 1.01) |
| Parent (11-15) | 13.56 (12.56, 14.57) | 15.05 (13.85, 16.26) | 15.13 (14.02, 16.25) | 1.49 (-0.69, 3.04) | 1.57 (0.08, 3.07)* | 0.08 (-1.56, 1.73) |
| Teacher (all ages) | 9.74 (8.80, 10.60) | 10.60 (9.44, 11.75) | 9.87 (8.63, 11.11) | 0.85 (-0.57, 2.28) | 0.13 (-1.34, 1.60) | -0.72 (-2.41, 0.96) |
| Teacher (5-10) | 9.02 (7.77, 10.27) | 9.35 (7.55, 11.15) | 10.20 (8.23, 12.16) | 0.33 (-1.91, 2.57) | 1.18 (-1.03, 3.39) | 0.85 (-1.86, 3.56) |
| Teacher (11-15) | 10.38 (9.18, 11.57) | 11.20 (9.72, 12.69) | 9.60 (7.99, 11.05) | 0.83 (-1.05, 2.70) | -0.77 (-2.75, 1.21) | -1.60 (-3.79, 0.59) |
| Self (11-15) | 15.62 (14.80, 16.44) | 15.23 (14.11, 16.34) | 16.34 (15.28, 17.40) | -0.40 (-1.75, 0.96) | 0.72 (-0.59, 2.03) | 1.11 (-0.41, 2.64) |
| **SDQ impact score** |  |  |  |  |  |  |
| Parent (all ages) | 1.53 (1.29, 1.76) | 2.81 (2.37, 3.26) | 3.19 (2.84, 3.55) | 1.29 (0.83, 1.75)*** | 1.67 (1.25, 2.08)*** | 0.38 (-0.18, 0.95) |
| Parent (5-10) | 1.49 (1.14, 1.84) | 2.76 (1.90, 3.62) | 3.21 (2.60, 3.84) | 1.27 (0.50, 2.04)*** | 1.72 (1.06, 2.38)*** | 0.46 (-0.57, 1.49) |
| Parent (11-15) | 1.55 (1.23, 1.88) | 2.84 (2.31, 3.36) | 3.18 (2.74, 3.62) | 1.29 (0.70, 1.87)*** | 1.63 (1.09, 2.17)*** | 0.34 (-0.34, 1.02) |
| Teacher (all ages) | 0.95 (0.76, 1.14) | 1.29 (0.99, 1.59) | 1.06 (0.80, 1.33) | 0.34 (-0.00, 0.68) | 0.12 (-0.21, 0.44) | -0.22 (-0.62, 0.18) |
| Teacher (5-10) | 0.73 (0.48, 0.98) | 1.00 (0.50, 1.50) | 0.91 (0.57, 1.25) | 0.27 (-0.23, 0.76) | 0.18 (-0.24, 0.59) | -0.09 (-0.66, 0.48) |
| Teacher (11-15) | 1.14 (0.85, 1.42) | 1.43 (1.05, 1.81) | 1.19 (0.79, 1.60) | 0.29 (-0.17, 0.75) | 0.05 (-0.43, 0.54) | -0.24 (-0.79, 0.31) |
| Self (11-15) | 1.07 (0.81, 1.34) | 1.80 (1.37, 2.24) | 2.25 (1.81, 2.68) | 0.73 (0.26, 1.21)** | 1.17 (0.69, 1.66)*** | 0.44 (-0.18, 1.06) |
| Note: All ages refers to 5-15 years. Impact scores when parent or self-reported range from 0 to 10, while teacher impact scores range from 0 to 6. Self-reports completed at 11-15 years only. Observations for children with any emotional disorder were n=130 in 1999, n=54 in 2004, and n=88 in 2017. For adolescents, observations were n=162 in 1999, n=118 in 2004, n=149 in 2017. Note these samples vary depending on informant. | | | | | | |

| **Table S9:** Comparison of difficulties of children (5-10 years) and adolescents (11-15 years) with any ICD-10 behavioural disorder | | | | | | |
| --- | --- | --- | --- | --- | --- | --- |
|  | Cohort of children with ICD-10 behavioural disorder | | | Survey Comparisons | | |
|  | 1999 | 2004 | 2017 | 1999 v 2004 | 1999 v 2017 | 2004 v 2017 |
| **SDQ total difficulty score (mean, 95% CI)** |  |  |  |  |  |  |
| Parent (all ages) | 17.41 (16.70, 18.11) | 17.57 (16.76, 18.37) | 19.42 (18.55, 20.30) | 0.16 (-0.90, 1.22) | 2.01 (0.89, 3.14)*** | 1.86 (0.66, 3.05)*** |
| Parent (5-10) | 17.57 (16.61, 18.53) | 18.11 (16.91, 19.30) | 19.10 (17.89, 20.31) | 0.54 (-0.97, 2.05) | 1.53 (0.01, 3.06)* | 0.99 (-0.70, 2.68) |
| Parent (11-15) | 17.24 (16.19, 18.28) | 17.13 (16.03, 18.22) | 19.82 (18.53, 21.11) | -0.11 (-1.62, 1.40) | 2.58 (0.91, 4.25)*** | 2.69 (0.98, 4.41)*** |
| Teacher (all ages) | 17.46 (16.62, 18.30) | 17.69 (16.79, 18.59) | 14.76 (13.51, 16.01) | 0.23 (-1.01, 1.46) | -2.70 (-4.19, -1.22)*** | -2.93 (-4.44, 1.42)*** |
| Teacher (5-10) | 17.17 (16.06, 18.28) | 18.00 (16.66, 19.34) | 14.80 (13.24, 16.35) | 0.83 (-0.91, 2.56) | -2.38 (-4.27, -0.49)* | -3.20 (-5.24, -1.17)** |
| Teacher (11-15) | 17.83 (16.54, 19.12) | 17.41 (16.18, 18.64) | 14.69 (12.52, 16.87) | -0.42 (-2.20, 1.36) | -3.14 (-5.58, -0.69)*** | -2.72 (-5.06, -0.37)*** |
| Self (11-15) | 14.64 (13.80, 15.49) | 16.12 (15.18, 17.08) | 15.56 (14.04, 17.09) | 1.48 (0.22, 2.75)*** | 0.92 (-0.69, 2.53) | -0.56 (-2.26, -1.13) |
| **SDQ impact score** |  |  |  |  |  |  |
| Parent (all ages) | 2.03 (1.80, 2.27) | 3.19 (2.86, 3.53) | 3.78 (3.42, 4.15) | 1.16 (0.76, 1.55)*** | 1.75 (1.34, 2,16)*** | 0.59 (0.10, 1.08)* |
| Parent (5-10) | 1.85 (1.53, 2.17) | 3.25 (2.73, 3.77) | 3.53 (3.03, 4.03) | 1.39 (0.82, 1.97)*** | 1.69 (1.11, 2.25)*** | 0.29 (-0.43, 1.00) |
| Parent (11-15) | 2.23 (1.90, 2.57) | 3.15 (2.71, 3.59) | 4.09 (3.57, 4.61) | 0.92 (0.37, 1.46)*** | 1.86 (1.27, 2.44)*** | 0.94 (0.25, 1.63)** |
| Teacher (all ages) | 2.21 (2.02, 2.41) | 2.46 (2.25, 2.68) | 2.21 (1.87, 2.56) | 0.25 (-0.04, 0.54) | 0.00 (-0.37, 0.37) | -0.25 (-0.64, 0.13) |
| Teacher (5-10) | 2.15 (1.88, 2.42) | 2.51 (2.19, 2.82) | 2.25 (1.81, 2.70) | 0.36 (-0.06, 0.77) | 0.10 (-0.39, 0.59) | -0.26 (-0.78, 0.27) |
| Teacher (11-15) | 2.29 (2.01, 2.58) | 2.42 (2.12, 2.73) | 2.14 (1.58, 2.71) | 0.13 (-0.29, 0.55) | -0.15 (-0.72, 0.42) | -0.28 (-0.87, 0.31) |
| Self (11-15) | 0.71 (0.51, 0.91) | 1.18 (0.88, 1.49) | 0.91 (0.54, 1.28) | 0.47 (0.12, 0.83)** | 0.20 (-0.19, 0.59) | -0.28 (-0.78, 0.22) |
| Note: All ages refers to 5-15 years. Impact scores when parent or self-reported range from 0 to 10, while teacher impact scores range from 0 to 6. Self-reports completed at 11-15 years only. Observations for children with any behavioural disorder were n=177 in 1999, n=127 in 2004, and n=120 in 2017. For adolescents, observations were n=167 in 1999, n=162 in 2004, n=99 in 2017. Note these samples vary depending on informant. | | | | | | |

| **Table S10:** Comparison of difficulties of children (5-10 years) and adolescents (11-15 years) with any ICD-10 hyperkinetic disorder | | | | | | |
| --- | --- | --- | --- | --- | --- | --- |
|  | Cohort of children with ICD-10 hyperkinetic disorder | | | Survey Comparisons | | |
|  | 1999 | 2004 | 2017 | 1999 v 2004 | 1999 v 2017 | 2004 v 2017 |
| **SDQ total difficulty score (mean, 95% CI)** |  |  |  |  |  |  |
| Parent (all ages) | 19.39 (18.29, 20.51) | 20.34 (18.96, 21.72) | 20.88 (19.72, 22.03) | 0.95 (-0.80, 2.69) | 1.48 (-0.12, 3.08) | 0.53 (-1.24, 2.31) |
| Parent (5-10) | 19.15 (17.83, 20.47) | 20.19 (18.50, 21.88) | 19.49 (17.94, 21.04) | 1.04 (-1.04, 3.13) | 0.34 (-1.66, 2.35) | -0.70 (-2.97, 1.56) |
| Parent (11-15) | 19.73 (17.76, 21.70) | 20.56 (18.10, 23.02) | 22.46 (20.81, 24.11) | 0.83 (-2.24, 3.90) | 2.73 (0.17, 5.29)* | 1.90 (-0.91, 4.71) |
| Teacher (all ages) | 19.39 (18.04, 20.73) | 17.04 (15.20, 18.88) | 17.79 (16.12, 19.45) | -2.35 (-4.60, 0.09) | -1.60 (-3.84, 0.64) | 0.75 (-1.73, 3.23) |
| Teacher (5-10) | 19.58 (18.05, 21.10) | 17.79 (15.66, 19.91) | 17.52 (15.38, 19.66) | -1.79 (-4.34, 0.76) | -2.06 (-4.67, 0.55) | -0.27 (-3.23, 2.69) |
| Teacher (11-15) | 19.10 (16.55, 21.65) | 15.80 (12.21, 19.39) | 18.22 (15.28, 21.16) | -3.30 (-7.60, 0.99) | -0.88 (-5.06, 3.30) | 2.42 (-2.12, 6.97) |
| Self (11-15) | 16.95 (15.11, 18.78) | 15.79 (13.65, 17.94) | 17.46 (14.82, 20.10) | -1.16 (-3.98, 1.67) | 0.51 (-2.54, 3.56) | 1.67 (-1.68, 5.02) |
| **SDQ impact score** |  |  |  |  |  |  |
| Parent (all ages) | 3.04 (2.59, 3.49) | 4.03 (3.37, 4.68) | 4.27 (3.74, 4.81) | 0.99 (0.22, 1.75)* | 1.23 (0.55, 1.92)*** | 0.25 (-0.58, 1.08) |
| Parent (5-10) | 3.15 (2.52, 3.78) | 4.00 (3.17, 4.83) | 3.83 (3.09, 4.57) | 0.85 (-0.16, 1.87) | 0.68 (-0.28, 1.64) | -0.17 (-1.27, 0.93) |
| Parent (11-15) | 2.89 (2.23, 3.54) | 4.06 (2.95, 5.18) | 4.78 (4.01, 5.56) | 1.17 (-0.02, 2.37) | 1.89 (0.90, 2.88)*** | 0.72 (-0.58, 2.01) |
| Teacher (all ages) | 2.83 (2.51, 3.14) | 2.46 (2.00, 2.92) | 3.00 (2.44, 3.56) | -0.37 (-0.90, 0.17) | 0.17 (-0.42, 0.76) | 0.54 (-0.17, 1.25) |
| Teacher (5-10) | 2.91 (2.50, 3.33) | 2.79 (2.25, 3.34) | 3.24 (2.56, 3.92) | -0.12 (-0.80, 0.56) | 0.33 (-0.42, 1.07) | 0.45 (0.40, 1.29) |
| Teacher (11-15) | 2.69 (2.19, 3.19) | 1.90 (1.08, 2.72) | 2.61 (1.57, 3.65) | -0.79 (-1.68, 0.10) | -0.08 (-1.07, 0.91) | 0.71 (-0.55, 1.97) |
| Self (11-15) | 0.55 (0.21, 0.89) | 1.00 (0.40, 1.60) | 0.88 (0.25, 1.51) | 0.45 (-0.17, 1.07) | 0.33 (-0.32, 0.97) | -0.12 (-0.97, 0.73) |
| Note: All ages refers to 5-15 years. Impact scores when parent or self-reported range from 0 to 10, while teacher impact scores range from 0 to 6. Self-reports completed at 11-15 years only. Observations for children with any hyperkinetic disorder were n=61 in 1999, n=47 in 2004, and n=47 in 2017. For adolescents, observations were n=45 in 1999, n=32 in 2004, n=41 in 2017. Note these samples vary depending on informant. | | | | | | |

| **Table S11:** Comparison of difficulties of children (5-10 years) and adolescents (11-15 years) with any ICD-10 comorbid disorder | | | | | | |
| --- | --- | --- | --- | --- | --- | --- |
|  | Cohort of children with ICD-10 comorbid disorder | | | Survey Comparisons | | |
|  | 1999 | 2004 | 2017 | 1999 v 2004 | 1999 v 2017 | 2004 v 2017 |
| **SDQ total difficulty score (mean, 95% CI)** |  |  |  |  |  |  |
| Parent (all ages) | 21.84 (20.19, 23.48) | 22.35 (20.65, 24.05) | 23.73 (22.72, 24.74) | 0.51 (-1.84, 2.87) | 1.89 (0.08, 3.71)* | 1.38 (-0.46, 3.22) |
| Parent (5-10) | 25.38 (23.44, 27.33) | 22.89 (19.90, 25.89) | 23.96 (22.54, 25.38) | -2.49 (-5.87, 0.89) | -1.42 (03.74, 0.90) | 1.07 (-1.79, 3.93) |
| Parent (11-15) | 19.22 (16.98, 21.46) | 21.98 (19.88, 24.07) | 23.55 (22.11, 24.99) | 2.76 (-0.30, 5.81) | 4.33 (1.82, 6.85)*** | 1.58 (-0.86, 4.02) |
| Teacher (all ages) | 16.27 (14.42, 18.12) | 18.76 (16.53, 20.98) | 15.93 (14.13, 17.73) | 2.49 (-0.36, 5.33) | -0.34 (-2.90, 2.22) | -2.83 (-5.63, 0.02) |
| Teacher (5-10) | 15.32 (12.31, 18.33) | 15.10 (11.07, 19.12) | 13.90 (11.30, 16.50) | -0.23 (-5.01, 4.56) | -1.42 (-5.29, 2.45) | -1.19 (-5.64, 3.26) |
| Teacher (11-15) | 17.03 (14.61, 19.45) | 21.50 (19.36, 23.64) | 17.58 (15.12, 20.04) | 4.47 (1.22, 7.72)** | 0.55 (-2.85, 3.95) | -3.92 (-7.27, -0.58)* |
| Self (11-15) | 16.86 (15.37, 18.36) | 18.56 (16.82, 20.32) | 18.93 (17.23, 20.64) | 1.70 (-0.58, 3.98) | 2.07 (-0.17, 4.31) | 0.37 (-2.13, 2.87) |
| **SDQ impact score** |  |  |  |  |  |  |
| Parent (all ages) | 3.75 (3.15, 4.35) | 5.51 (4.74, 6.29) | 5.82 (5.31, 6.32) | 1.76 (0.81, 2.72)*** | 2.07 (1.29, 2.84)*** | 0.31 (-0.58, 1.18) |
| Parent (5-10) | 4.15 (3.13, 5.16) | 5.21 (3.96, 6.46) | 5.50 (4.76, 6.23) | 1.06 (-0.49, 2.63) | 1.35 (0.14, 2.57)* | 0.29 (-1.06, 1.63) |
| Parent (11-15) | 3.46 (2.71, 4.20) | 5.73 (4.69, 6.76) | 6.06 (5.37, 6.75) | 2.27 (1.04, 3.50)*** | 2.60 (1.58, 3.63)*** | 0.33 (-0.84, 1.51) |
| Teacher (all ages) | 1.81 (1.41, 2.21) | 2.71 (2.19, 3.24) | 2.35 (1.87, 2.83) | 0.90 (0.26, 1.55)** | 0.54 (-0.09, 1.17) | -0.36 (-1.08, 0.35) |
| Teacher (5-10) | 1.61 (0.99 2.23) | 1.90 (1.08, 2.73) | 2.03 (1.30, 2.76) | 0.30 (-0.68, 1.28) | 0.42 (-0.52, 1.37) | 0.13 (-0.96, 1.22) |
| Teacher (11-15) | 1.97 (1.42, 2.53) | 3.32 (2.71, 3.94) | 2.60 (1.95, 3.27) | 1.35 (0.54, 2.16)** | 0.63 (-0.22, 1.49) | -0.72 (-1.63, 0.20) |
| Self (11-15) | 1.41 (0.81, 2.01) | 2.23 (1.48, 2.99) | 2.11 (1.42, 2.80) | 0.82 (-0.12, 1.76) | 0.70 (-0.21, 1.60) | -0.12 (-1.16, 0.91) |
| Note: All ages refers to 5-15 years. Impact scores when parent or self-reported range from 0 to 10, while teacher impact scores range from 0 to 6. Self-reports completed at 11-15 years only. Observations for children with any comorbid disorder were n=34 in 1999, n=28 in 2004, and n=52 in 2017. For adolescents, observations were n=45 in 1999, n=32 in 2004, n=41 in 2017. Note these samples vary depending on informant. | | | | | | |

| **Table 12**: **Comparison of sociodemographic and family characteristics of children with DAWBA-identified psychiatric disorder (univariable models)** | | | | | | | | | | | | |
| --- | --- | --- | --- | --- | --- | --- | --- | --- | --- | --- | --- | --- |
|  | 1999 (n=8,772) | | | 2004 (n=6,498) | | | 2017 (n=6,219) | | | Survey comparisons | | |
|  | With disorder  (n=769) | Without disorder  (n=8,003) | OR  (95% CI) | With disorder  (n=577) | Without disorder  (n=5,921) | OR  (95% CI) | With disorder  (n=619) | Without disorder  (n=5,600) | OR  (95% CI) | 1999 v 2004  (OR,  (95% CI) | 1999 v 2017  (OR,  (95% CI) | 2004 v 2017  (OR,  (95% CI) |
| Age (11-15 years %)† | 51.37 | 42.02 | 1.46 (1.26, 1.69)*** | 59.10 | 45.67 | 1.72 (1.44, 2.05)*** | 53.31 | 40.93 | 1.65 (1.40, 1.95)*** | 1.18 (0.94, 1.48) | 1.06 (0.95, 1.19) | 0.96 (0.75, 1.22) |
| Female (%) | 39.79 | 51.22 | 0.63 (0.54, 0.73)*** | 39.17 | 49.03 | 0.67 (0.56, 0.80)*** | 43.13 | 50.45 | 0.75 (0.63, 0.88)*** | 1.06 (0.84, 1.34) | 1.09 (0.97, 1.22) | 1.11 (0.87, 1.42) |
| From ethnic minority background (%) | 9.11 | 9.94 | 0.91 (0.70, 1.17) | 10.4 | 14.79 | 0.67 (0.51, 0.88)** | 10.2 | 22.12 | 0.39 (0.30, 0.51)*** | 0.74 (0.50, 1.07) | 0.66 (0.55, 0.79)*** | 0.59 (0.40, 0.86)** |
| In rented accommodation  (%) | 9.11 | 6.03 | 1.91 (1.72, 2.13)*** | 11.44 | 7.10 | 1.79 (1.59, 2.01)*** | 20.10 | 18.63 | 1.36 (1.23, 1.50)*** | 0.93 (0.80, 1.09) | 0.84 (0.78, 0.91)*** | 0.76 (0.65, 0.89)*** |
| Lowest income quintile (%) | 39.36 | 21.51 | 2.37 (2.02, 2.78)*** | 32.99 | 19.13 | 2.08 (1.70, 2.54)*** | 32.47 | 22.64 | 1.64 (1.36, 1.99)*** | 0.88 (0.68, 1.13) | 0.83 (0.74, 0.94)** | 0.79 (0.60, 1.04) |
| Neither parent working (%) | 27.23 | 12.08 | 1.61 (1.47, 1.77)*** | 33.86 | 14.13 | 1.77 (1.60, 1.96)*** | 27.60 | 9.45 | 1.81 (1.63, 2.01*** | 1.09 (0.96, 1.25) | 1.06 (0.99, 0.11) | 1.02 (0.88, 1.19) |
| Lone parent (%) | 38.75 | 20.72 | 2.42 (2.07, 2.83)*** | 41.94 | 22.58 | 2.48 (2.08, 2.95)*** | 38.45 | 19.52 | 2.58 (2.16, 3.07)*** | 1.02 (0.81, 1.29) | 1.03 (0.92, 1.16) | 1.04 (0.81, 1.33) |
| Unhealthy family functioning (%) | 35.46 | 17.16 | 2.65 (2.26, 3.11)*** | 34.05 | 15.68 | 2.78 (2.30, 3.36)*** | 31.29 | 14.72 | 2.64 (2.18, 3.19)*** | 1.05 (0.82, 1.34) | 0.99 (0.88, 1.13) | 0.95 (0.73, 1.24) |
| Parent has mental health problem (%) | 46.33 | 23.03 | 2.89 (2.48, 3.36)*** | 47.76 | 20.02 | 3.65 (3.06, 4.37)*** | 40.88 | 16.69 | 3.45 (2.89, 4.13)*** | 1.27 (0.99, 1.60) | 1.09 (0.97, 1.23) | 0.95 (0.73, 1.22) |
| Note: ****p* < 0.001. ***p* < 0.01. *p<0.05, †reference category 5 to 10 year olds  Sample sizes vary from n=8,205-8,772 for the 1999 survey, from n=5,827-6,498 for the 2004 survey, and from 5,590-6,219 for the 2017 survey | | | | | | | | | | | | |

| **Table S13**: Comparison of characteristics of children with any emotional disorder (all ages) | | | | | | | | | | | | |
| --- | --- | --- | --- | --- | --- | --- | --- | --- | --- | --- | --- | --- |
|  | 1999 (n=8,722) | | | 2004 (n=6,498) | | | 2017 (n=6,219) | | | Survey Comparisons | | |
|  | With disorder  (n=292) | Without disorder  (n=8,480) | OR  (95% CI) | With disorder  (n=172) | Without disorder  (n=6,326) | OR  (95% CI) | With disorder  (n=237) | Without disorder  (n=5,982) | OR  (95% CI) | 1999 v 2004 (OR,  (95% CI) | 1999 v 2017 (OR,  (95% CI) | 2004 v 2017 (OR,  (95% CI) |
| Age (11-15 years %) | 55.48 | 42.41 | 1.69 (1.34, 2.14)*** | 68.60 | 46.27 | 2.54 (1.83, 3.51)*** | 62.87 | 41.34 | 2.40 (1.84, 3.14)*** | 1.05 (1.01, 1.08)* | 1.04 (1.02, 1.06)*** | 1.04 (1.01, 1.06)*** |
| Female (%) | 55.82 | 50.02 | 1.26 (1.00, 1.60)* | 62.21 | 47.77 | 1.80 (1.32, 2.46)*** | 57.38 | 49.41 | 1.38 (1.06, 1.79)* | 1.43 (0.96, 2.11) | 1.04 (0.88, 1.25) | 0.77 (0.51, 1.15) |
| From ethnic minority background (%) | 11.00 | 9.83 | 1.13 (0.78, 1.65) | 11.63 | 14.47 | 0.78 (0.49, 1.25) | 13.50 | 21.20 | 0.58 (0.40, 0.85)* | 0.69 (0.38, 1.25) | 0.72 (0.55, 0.93)* | 0.75 (0.41, 1.36) |
| In rented accommodation (%) | 8.59 | 6.22 | 1.50 (1.27, 1.78)*** | 13.95 | 7.31 | 1.73 (1.42, 2.12)*** | 26.27 | 18.48 | 1.39 (1.20, 1.62)** | 1.15 (0.88, 1.50) | 0.96 (0.86, 1.08) | 0.80 (0.62, 1.04) |
| Lowest income quintile (%) | 32.21 | 22.76 | 1.61 (1.24, 2.09)*** | 28.37 | 20.10 | 1.57 (1.09, 2.28)*** | 30.95 | 23.31 | 1.47 (1.09, 1.99)*** | 0.98 (0.62, 1.54) | 0.96 (0.78, 1.17) | 0.94 (0.58, 1.51) |
| Neither parent working (%) | 23.88 | 13.05 | 1.45 (1.25, 1.67)*** | 30.00 | 15.52 | 1.52 (1.28, 1.82)*** | 22.27 | 10.80 | 1.47 (1.24, 1.74)*** | 1.05 (0.84, 1.32) | 1.01 (0.90, 1.13) | 0.97 (0.76, 1.24) |
| Lone parent (%) | 33.90 | 21.90 | 1.83 (1.43, 2.34)*** | 43.60 | 23.77 | 2.48 (1.82, 3.37)*** | 39.24 | 20.70 | 2.47 (1.89, 3.24)*** | 1.36 (0.91, 2.01) | 1.16 (0.97, 1.40) | 1.00 (0.66, 1.50) |
| Unhealthy family functioning (%) | 29.58 | 18.39 | 1.86 (1.43, 2.42)*** | 26.67 | 17.05 | 1.77 (1.24, 2.51)*** | 24.77 | 15.99 | 1.73 (1.27, 2.36)*** | 0.95 (0.61, 1.47) | 0.96 (0.79, 1.18) | 0.98 (0.61, 1.57) |
| Parent has mental health problem (%) | 46.53 | 24.35 | 2.71 (2.13, 3.43)*** | 54.55 | 21.61 | 4.35 (3.19, 5.95)*** | 38.22 | 18.30 | 2.76 (2.09, 3.64)*** | 1.61 (1.09, 2.38)* | 1.01 (0.84, 1.21) | 0.63 (0.42, 0.96)* |

| **Table S14**: Comparison of characteristics of children with any emotional disorder (11-15 years) | | | | | | | | | | | | |
| --- | --- | --- | --- | --- | --- | --- | --- | --- | --- | --- | --- | --- |
|  | 1999 (n=3,758) | | | 2004 (n=3,045) | | | 2017 (n=2,622) | | | Survey Comparisons | | |
|  | With disorder  (n=162) | Without disorder  (n=3,596) | OR  (95% CI) | With disorder  (n=118) | Without disorder  (n=2,927) | OR  (95% CI) | With disorder  (n=149) | Without disorder  (n=2,473) | OR  (95% CI) | 1999 v 2004 (OR,  (95% CI) | 1999 v 2017 (OR,  (95% CI) | 2004 v 2017 (OR,  (95% CI) |
| Female (%) | 57.41 | 49.00 | 1.40 (1.02, 1.93)* | 61.02 | 47.39 | 1.74 (1.19, 2.53)** | 61.74 | 49.41 | 1.65 (1.18, 2.32)** | 1.24 (0.76, 2.03) | 1.09 (0.86, 1.34) | 0.95 (0.57, 1.58) |
| From ethnic minority background (%) | 11.1 | 10.24 | 1.10 (0.66, 1.81) | 11.02 | 13.68 | 0.78 (0.44, 1.40) | 10.74 | 21.16 | 0.45 (0.26, 0.76)** | 0.71 (0.33, 1.54) | 0.64 (0.44, 0.92)* | 0.57 (0.26, 1.26) |
| In rented accommodation (%) | 5.59 | 5.65 | 1.51 (1.20, 1.92)*** | 16.10 | 6.32 | 2.05 (1.60, 2.61)*** | 22.97 | 15.22 | 1.34 (1.10, 1.64)** | 1.35 (0.96, 1.90) | 0.94 (0.81, 1.10) | 0.66 (0.48, 0.90)** |
| Lowest income quintile (%) | 33.33 | 20.87 | 1.90 (1.33, 2.70)*** | 28.42 | 18.46 | 1.57 (1.09, 2.28)*** | 29.46 | 23.83 | 1.47 (1.09, 1.99)*** | 0.98 (0.62, 1.54) | 0.96 (0.78, 1.17) | 0.94 (0.58, 1.51) |
| Neither parent working (%) | 20.75 | 12.34 | 1.37 (1.12, 1.67)** | 28.45 | 14.54 | 1.50 (1.20, 1.87)*** | 20.98 | 11.08 | 1.37 (1.10, 1.70)** | 1.10 (0.82, 1.48) | 1.00 (0.86, 1.16) | 0.91 (0.67, 1.24) |
| Lone parent (%) | 35.19 | 22.75 | 1.84 (1.32, 2.57)*** | 44.07 | 24.39 | 2.44 (1.68, 3.55)*** | 36.24 | 22.00 | 2.02 (1.42, 2.85)*** | 1.32 (0.80, 2.18) | 1.05 (0.82, 1.33) | 0.83 (0.50, 1.37) |
| Unhealthy family functioning (%) | 26.92 | 20.31 | 1.45 (1.01, 2.08)* | 28.57 | 18.18 | 1.80 (1.18, 2.74)** | 26.28 | 17.54 | 1.68 (1.13, 2.49)* | 1.25 (0.71, 2.17) | 1.08 (0.82, 1.41) | 0.93 (0.52, 1.66) |
| Parent has mental health problem (%) | 51.57 | 27.07 | 2.87 (2.08, 3.95)*** | 55.36 | 22.79 | 4.20 (2.86, 6.16)*** | 35.71 | 19.31 | 2.32 (1.62, 3.33)*** | 1.46 (0.89, 2.41) | 0.90 (0.71, 1.14) | 0.55 (0.33, 0.93)* |

| **Table S15**: Comparison of characteristics of children with any behavioural disorder (all ages) | | | | | | | | | | | | |
| --- | --- | --- | --- | --- | --- | --- | --- | --- | --- | --- | --- | --- |
|  | 1999 (n=8,722) | | | 2004 (n=6,498) | | | 2017 (n=6,219) | | | Survey Comparisons | | |
|  | With disorder  (n=344) | Without disorder  (n=8,428) | OR  (95% CI) | With disorder  (n=291) | Without disorder  (n=6,207) | OR  (95% CI) | With disorder  (n=219) | Without disorder  (n=6,000) | OR  (95% CI) | 1999 v 2004 (OR,  (95% CI) | 1999 v 2017 (OR,  (95% CI) | 2004 v 2017 (OR,  (95% CI) |
| Age (11-15 years %) | 48.55 | 42.61 | 1.27 (1.02, 1.58)*** | 55.67 | 46.45 | 1.45 (1.14, 1.83)*** | 45.21 | 42.05 | 1.14 (0.87, 1.49) | 1.14 (0.83, 1.57) | 0.95 (0.80, 1.12) | 0.79 (0.55, 1.13) |
| Female (%) | 29.65 | 51.06 | 0.40 (0.32, 0.51)*** | 28.87 | 49.06 | 0.42 (0.33 0.55)*** | 33.33 | 50.32 | 0.49 (0.37, 0.66)*** | 1.04 (0.74, 1.48) | 1.11 (0.92, 1.33) | 1.17 (0.80, 1.72) |
| From ethnic minority background (%) | 9.01 | 9.90 | 0.90 (0.62, 1.31) | 9.28 | 14.64 | 0.60 (0.40, 0.89)* | 8.22 | 21.37 | 0.33 (0.20, 0.54)*** | 0.66 (0.38, 1.15) | 0.60 (0.44, 0.82)*** | 0.55 (0.29, 1.04) |
| In rented accommodation (%) | 9.59 | 6.16 | 2.18 (1.87, 2.52)*** | 8.25 | 7.45 | 1.66 (1.41, 1.94)*** | 16.97 | 18.84 | 1.40 (1.19, 1.64)*** | 0.76 (0.61, 0.94)* | 0.80 (0.72, 0.89)*** | 0.84 (0.67, 1.06) |
| Lowest income quintile (%) | 42.07 | 22.28 | 2.53 (2.02, 3.17)*** | 34.66 | 19.66 | 2.17 (1.66, 2.84)*** | 34.57 | 23.21 | 1.75 (1.29, 2.38)*** | 0.86 (0.60, 1.22) | 0.83 (0.69, 1.00)* | 0.80 (0.54, 1.21) |
| Neither parent working (%) | 29.53 | 12.75 | 1.60 (1.41, 1.82)*** | 33.80 | 15.06 | 1.78 (1.55, 2.04)*** | 32.71 | 10.45 | 1.92 (1.63, 2.26)*** | 1.11 (0.92, 1.34) | 1.09 (0.99, 1.22) | 1.08 (0.87, 1.34) |
| Lone parent (%) | 41.57 | 21.51 | 2.60 (2.08, 3.24)*** | 39.18 | 23.60 | 2.08 (1.64, 2.66)*** | 39.27 | 20.75 | 2.47 (1.87, 3.26)*** | 0.80 (0.58, 1.11) | 0.98 (0.82, 1.16) | 1.18 (0.82, 1.71) |
| Unhealthy family functioning (%) | 40.36 | 17.88 | 3.11 (2.48, 3.89)*** | 38.87 | 16.28 | 3.27 (2.55, 4.19)*** | 31.43 | 15.78 | 2.45 (1.81, 3.30)*** | 1.05 (0.75, 1.47) | 0.89 (0.74, 1.07) | 0.75 (0.51, 1.11) |
| Parent has mental health problem (%) | 45.16 | 24.26 | 2.57 (2.07, 3.20)*** | 43.82 | 21.47 | 2.85 (2.24, 3.64)*** | 38.10 | 18.35 | 2.74 (2.06, 3.64)*** | 1.11 (0.80, 1.54) | 1.03 (0.86, 1.24) | 0.96 (0.66, 1.40) |

| **Table S16:** Comparison of characteristics of children with any hyperkinetic disorder (all ages) | | | | | | | | | | | | |
| --- | --- | --- | --- | --- | --- | --- | --- | --- | --- | --- | --- | --- |
|  | 1999 (n=8,722) | | | 2004 (n=6,498) | | | 2017 (n=6,219) | | | Survey Comparisons | | |
|  | With disorder  (n=106) | Without disorder  (n=8,294) | OR  (95% CI) | With disorder  (n=79) | Without disorder  (n=6,179) | OR  (95% CI) | With disorder  (n=88) | Without disorder  (n=5,775) | OR  (95% CI) | 1999 v 2004 (OR,  (95% CI) | 1999 v 2017 (OR,  (95% CI) | 2004 v 2017 (OR,  (95% CI) |
| Age (11-15 years %) | 42.45 | 42.26 | 1.01 (0.68, 1.49) | 40.51 | 46.20 | 0.79 (0.50, 1.25) | 46.59 | 40.95 | 1.26 (0.82, 1.92) | 0.79 (0.43, 1.43) | 1.12 (0.84, 1.49) | 1.59 (0.85, 2.94) |
| Female (%) | 16.98 | 50.60 | 0.20 (0.12, 0.33)*** | 8.86 | 48.36 | 0.10 (0.05, 0.23)*** | 22.73 | 50.03 | 0.29 (0.18, 0.49)*** | 0.52 (0.21, 1.32) | 1.21 (0.85, 1.73) | 2.83 (1.12, 7.14)* |
| From ethnic minority background (%) | 1.89 | 9.95 | 0.17 (0.04, 0.71)* | 2.53 | 14.66 | 0.15 (0.04, 0.62)** | 6.82 | 21.72 | 0.26 (0.11, 0.61)** | 0.87 (0.12, 6.32) | 1.23 (0.55, 2.78) | 1.74 (0.34, 8.92) |
| In rented accommodation (%) | 13.21 | 6.12 | 2.00 (1.54, 2.59)*** | 15.19 | 7.12 | 1.92 (1.44, 2.56)*** | 21.84 | 18.50 | 1.41 (1.10, 1.81)** | 0.96 (0.65, 1.42) | 0.84 (0.70, 1.00) | 0.73 (0.50, 1.07) |
| Lowest income quintile (%) | 42.57 | 27.95 | 2.20 (1.47, 3.29)*** | 32.86 | 19.61 | 2.01 (1.21, 3.17)*** | 37.66 | 27.10 | 1.52 (0.93, 2.46) | 0.91 (0.48, 1.74) | 0.83 (0.61, 1.14) | 0.76 (0.38, 1.52) |
| Neither parent working (%) | 23.58 | 12.71 | 1.51 (1.20, 1.91)*** | 38.46 | 14.82 | 1.88 (1.46, 2.43)*** | 30.23 | 10.07 | 1.81 (1.40, 2.36)*** | 1.25 (0.88, 1.76) | 1.10 (0.92, 1.31) | 0.96 (0.67, 1.39) |
| Lone parent (%) | 38.61 | 22.27 | 1.88 (1.25, 2.81)** | 32.86 | 19.61 | 2.14 (1.35, 3.37)*** | 31.17 | 23.01 | 2.05 (1.31, 3.20)*** | 1.14 (0.62, 2.09) | 1.05 (0.77, 1.41) | 0.96 (0.51, 1.82) |
| Unhealthy family functioning (%) | 35.85 | 17.86 | 2.57 (1.72, 3.84)*** | 32.47 | 16.55 | 2.42 (1.50, 3.93)*** | 29.76 | 15.21 | 2.36 (1.47, 3.79)*** | 0.94 (0.50, 1.77) | 0.96 (0.70, 1.31) | 0.97 (0.50, 1.91) |
| Parent has mental health problem (%) | 39.62 | 23.80 | 2.10 (1.42, 3.11)*** | 38.96 | 20.94 | 2.41 (1.52, 3.83)*** | 31.76 | 17.39 | 2.21 (1.39, 3.51)*** | 1.15 (0.63, 2.10) | 1.03 (0.76, 1.39) | 0.92 (0.48, 1.76) |

| **Table S17**: Comparison of characteristics of children with any cross-comorbid disorder (all ages) | | | | | | | | | | | | |
| --- | --- | --- | --- | --- | --- | --- | --- | --- | --- | --- | --- | --- |
|  | 1999 (n=8,722) | | | 2004 (n=6,498) | | | 2017 (n=6,219) | | | Survey Comparisons | | |
|  | With disorder  (n=80) | Without disorder  (n=8,692) | OR  (95% CI) | With disorder  (n=68) | Without disorder  (n=6,430) | OR  (95% CI) | With disorder  (n=119) | Without disorder  (n=6,100) | OR  (95% CI) | 1999 v 2004 (OR,  (95% CI) | 1999 v 2017 (OR,  (95% CI) | 2004 v 2017 (OR,  (95% CI) |
| Age (11-15 years %) | 57.50 | 42.71 | 1.82 (1.16, 2.83)*** | 58.82 | 46.73 | 1.63 (1.00, 2.65)*** | 56.30 | 41.89 | 1.79 (1.24, 2.58)*** | 0.90 (0.46, 1.73) | 0.99 (0.74, 1.32) | 1.10 (0.60, 2.02) |
| Female (%) | 33.75 | 50.37 | 0.50 (0.32, 0.80)*** | 39.71 | 48.24 | 0.71 (0.43, 1.15) | 39.50 | 49.92 | 0.65 (0.45, 0.95)* | 1.41 (0.72, 2.76) | 1.14 (0.85, 1.54) | 0.93 (0.50, 1.71) |
| From ethnic minority background (%) | 7.50 | 9.89 | 0.74 (0.32, 1.70) | 11.76 | 14.43 | 0.79 (0.38, 1.66) | 6.72 | 21.19 | 0.27 (0.13, 0.55)*** | 1.07 (0.35, 3.27) | 0.60 (0.35, 1.05) | 0.34 (0.12, 0.95)* |
| In rented accommodation (%) | 7.50 | 6.29 | 1.94 (1.44, 2.61)*** | 14.71 | 7.41 | 2.08 (1.53, 2.82)*** | 15.13 | 18.85 | 1.21 (0.97, 1.50) | 1.07 (0.70, 1.64) | 0.79 (0.66, 0.95)* | 0.58 (0.40, 0.84)** |
| Lowest income quintile (%) | 52.70 | 22.80 | 3.78 (2.38, 5.97)*** | 50.85 | 19.99 | 4.14 (2.48, 6.93)*** | 32.67 | 23.43 | 1.59 (1.04, 2.42)*** | 1.10 (0.55, 2.19) | 0.65 (0.47, 0.89)** | 0.38 (0.20, 0.74)** |
| Neither parent working (%) | 33.75 | 13.22 | 2.02 (1.56, 2.60)*** | 51.47 | 15.52 | 2.49 (1.89, 3.28)*** | 32.20 | 10.81 | 2.08 (1.68, 2.59)*** | 1.24 (0.85, 1.80) | 1.02 (0.86, 1.20) | 0.84 (0.59, 1.19) |
| Lone parent (%) | 46.25 | 22.08 | 3.04 (1.95, 4.72)*** | 55.88 | 23.97 | 4.02 (2.48, 6.51)*** | 37.82 | 21.08 | 2.28 (1.56, 3.31)*** | 1.32 (0.69, 2.55) | 0.87 (0.65, 1.16) | 0.57 (0.31, 1.04) |
| Unhealthy family functioning (%) | 49.37 | 18.48 | 4.30 (2.76, 6.71)*** | 46.03 | 17.01 | 4.16 (2.52, 6.86)*** | 45.13 | 15.77 | 4.39 (3.01, 6.41)*** | 0.97 (0.50, 1.89) | 1.01 (0.76, 1.35) | 1.06 (0.56, 1.97) |
| Parent has mental health problem (%) | 60.00 | 24.76 | 4.56 (2.91, 7.15)*** | 63.08 | 22.05 | 6.04 (3.64, 10.03)*** | 53.98 | 18.37 | 5.21 (3.58, 7.59)*** | 1.32 (0.67, 2.61) | 1.07 (0.80, 1.43) | 0.86 (0.46, 1.62) |


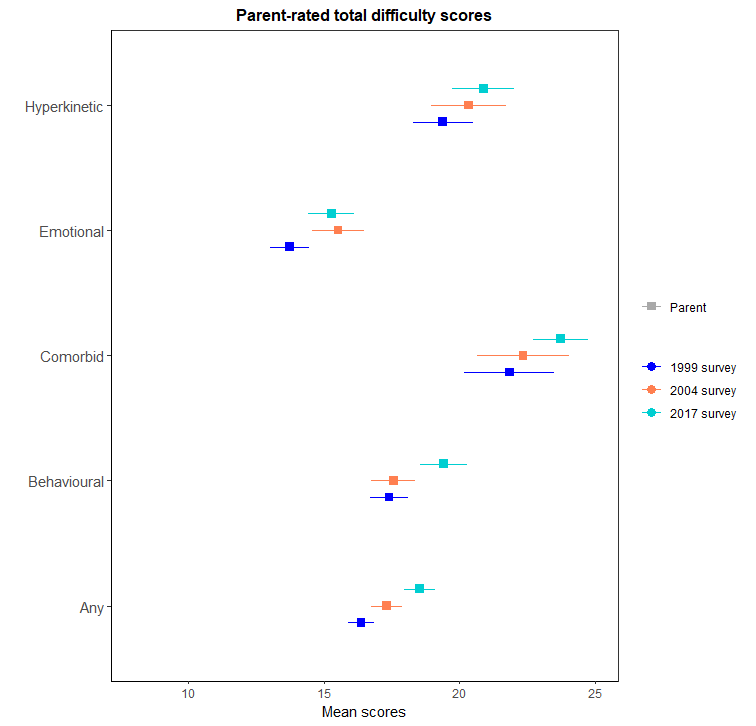

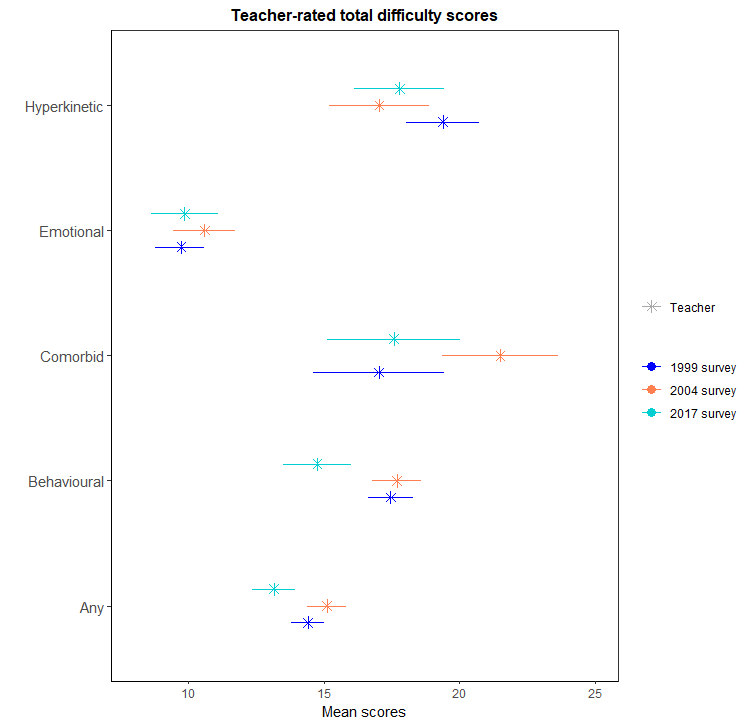


**Figure S1**: Cross survey comparison of parent- and teacher-rated mean total difficulty scores (SDQ) among those aged 5-15 years


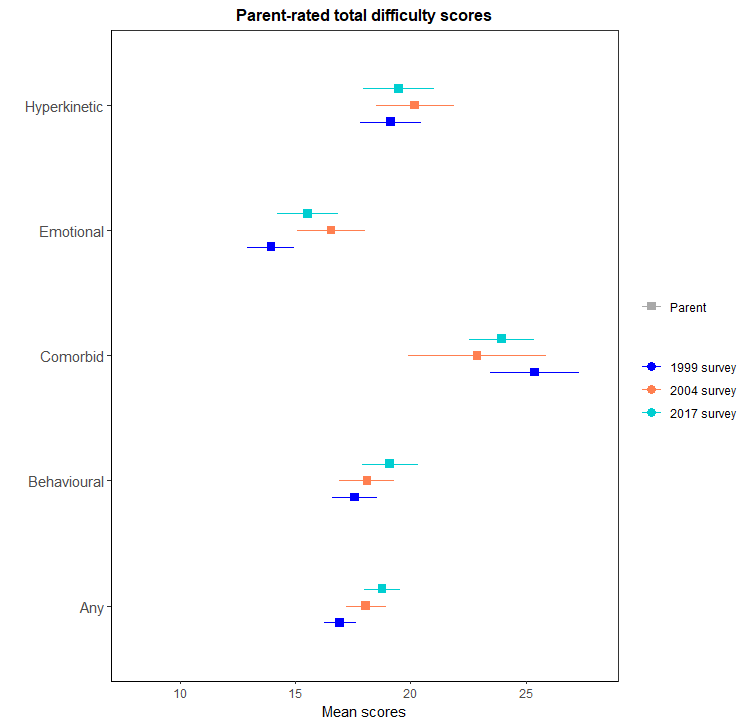


**Figure S2**: Cross survey comparison of parent- and teacher-rated mean total difficulty scores (SDQ) among those aged 5-10 years


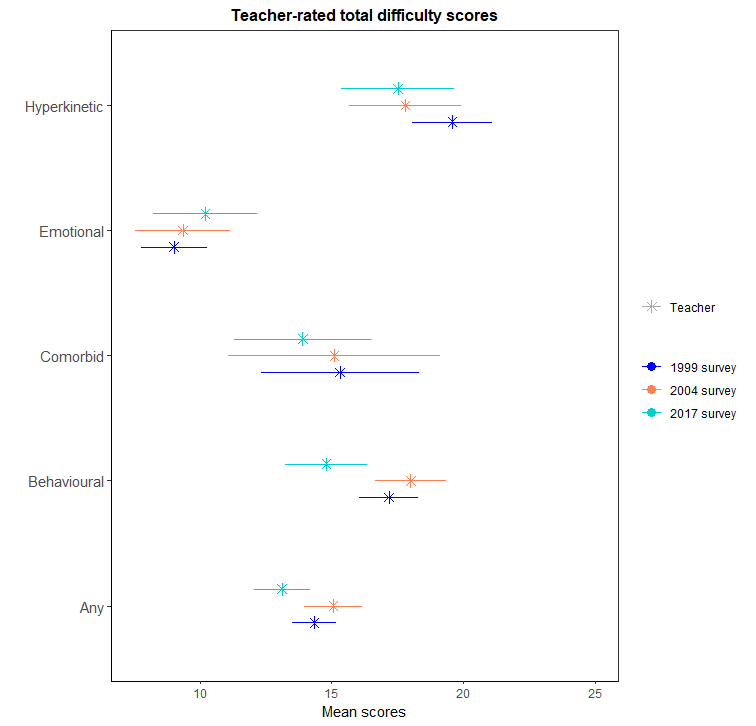


**Figure S3**: Cross survey comparison of parent and teacher-rated impact scores among those aged 5-15 years. Note impact scores for parent reports range from 0-10, and for teacher reports from 0-6.


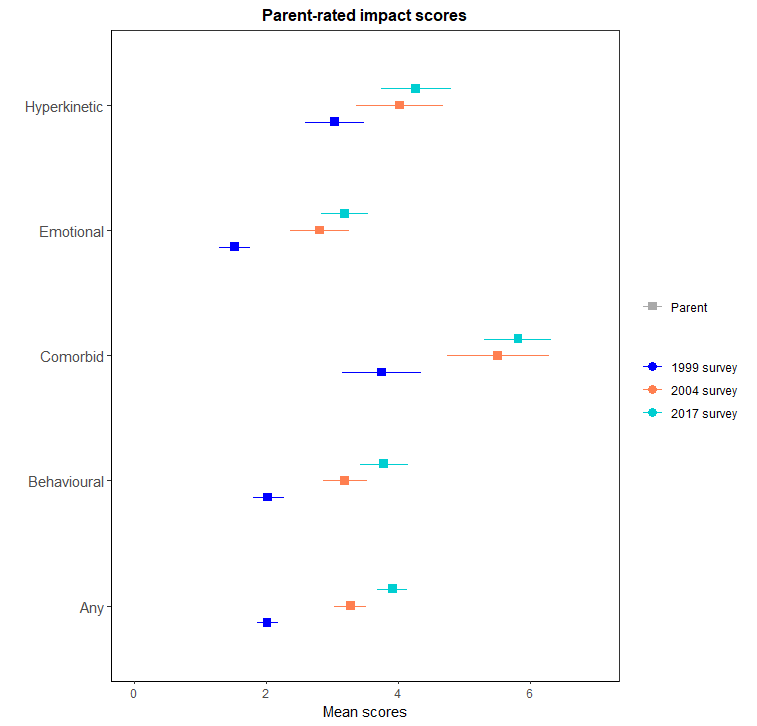

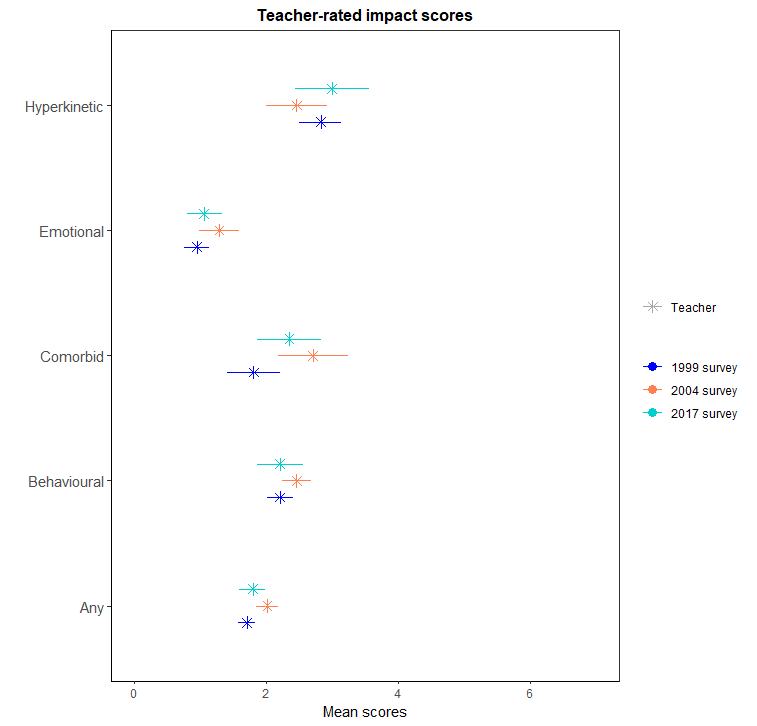


**Figure S4**: Cross survey comparison of parent and teacher-rated impact scores among those aged 5-10 years. Note impact scores for parent reports range from 0-10, and for teacher reports from 0-6.


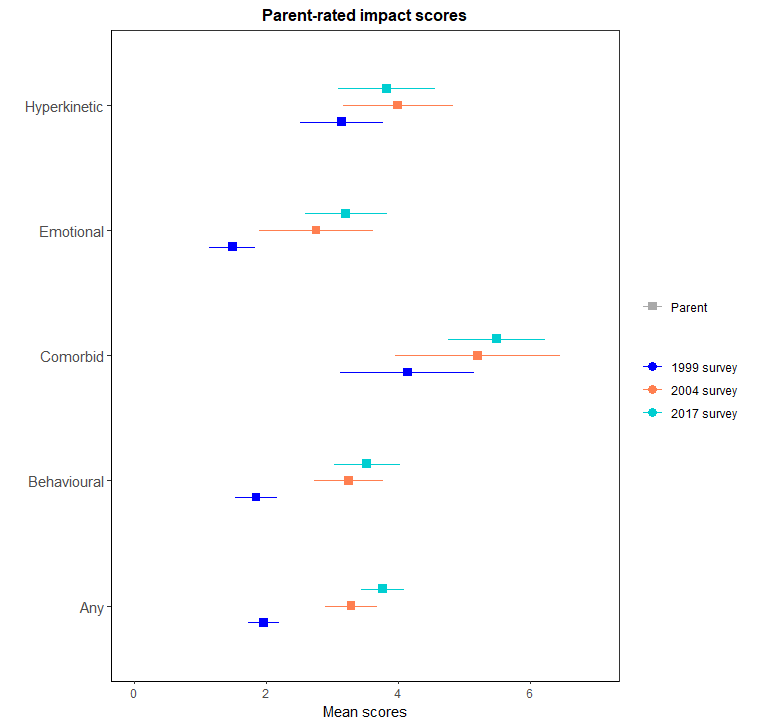

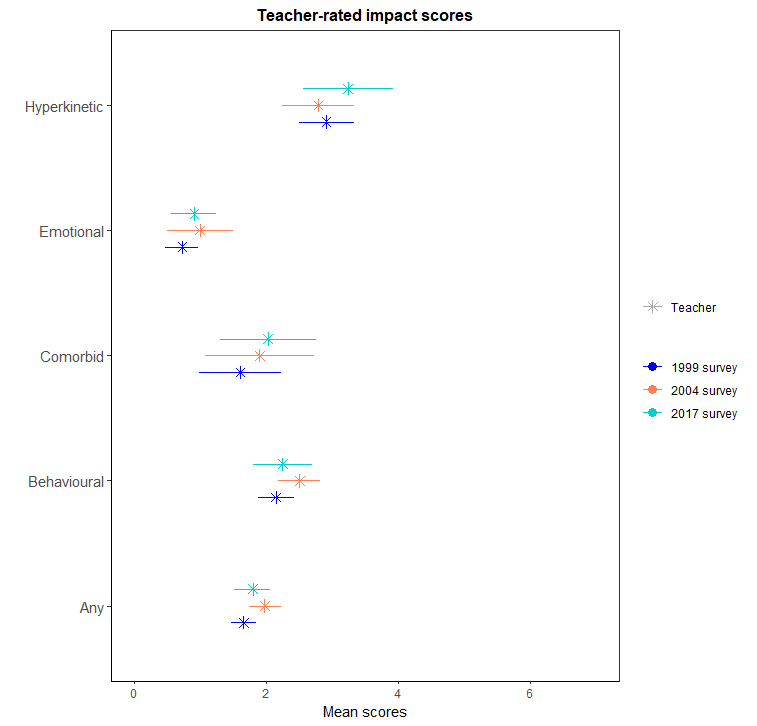


**Figure S5**: Odds of children having any emotional disorder based on sociodemographic and family characteristics


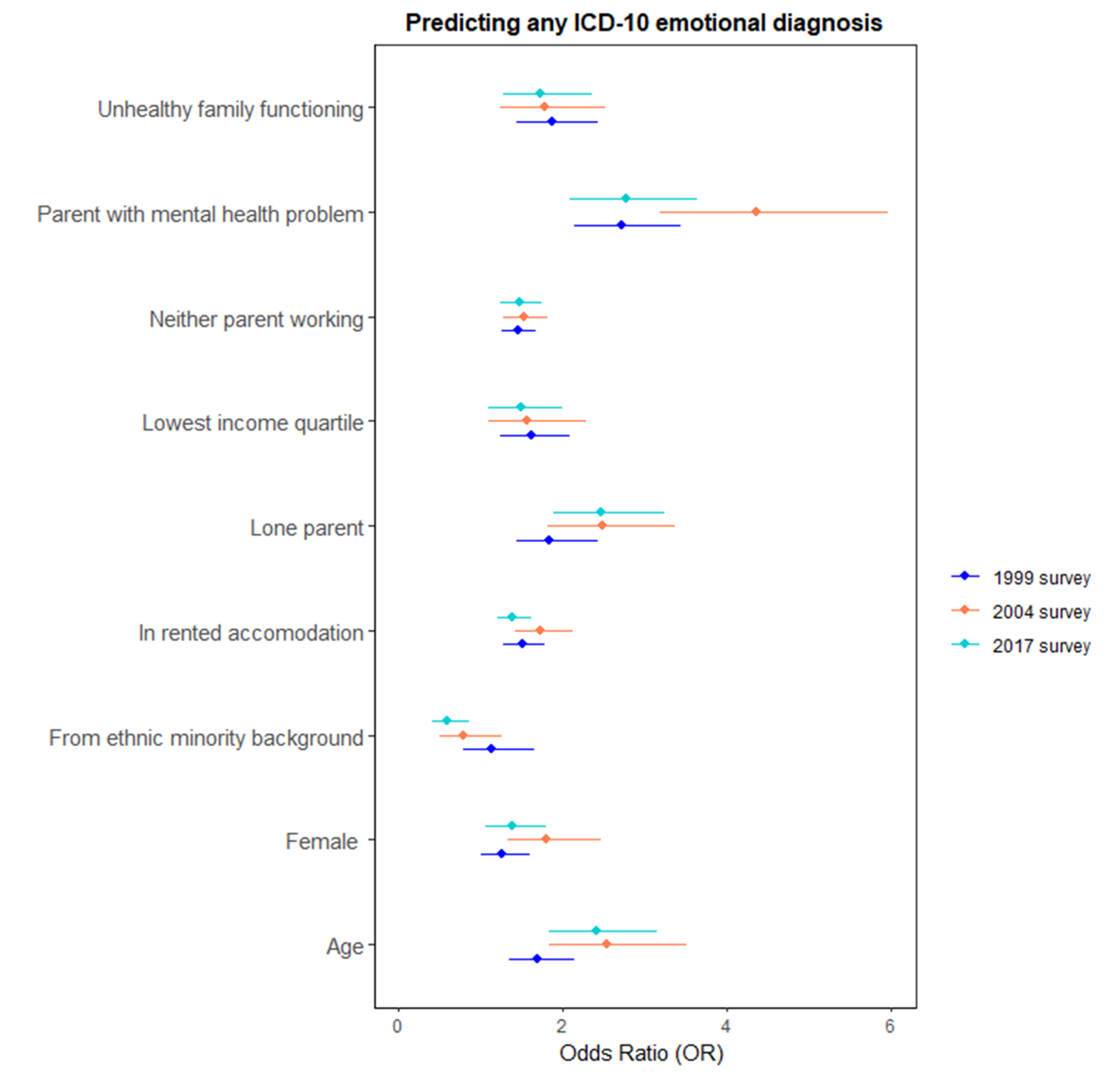


**Figure S6**: Odds of children having any behavioural disorder based on sociodemographic and family characteristics


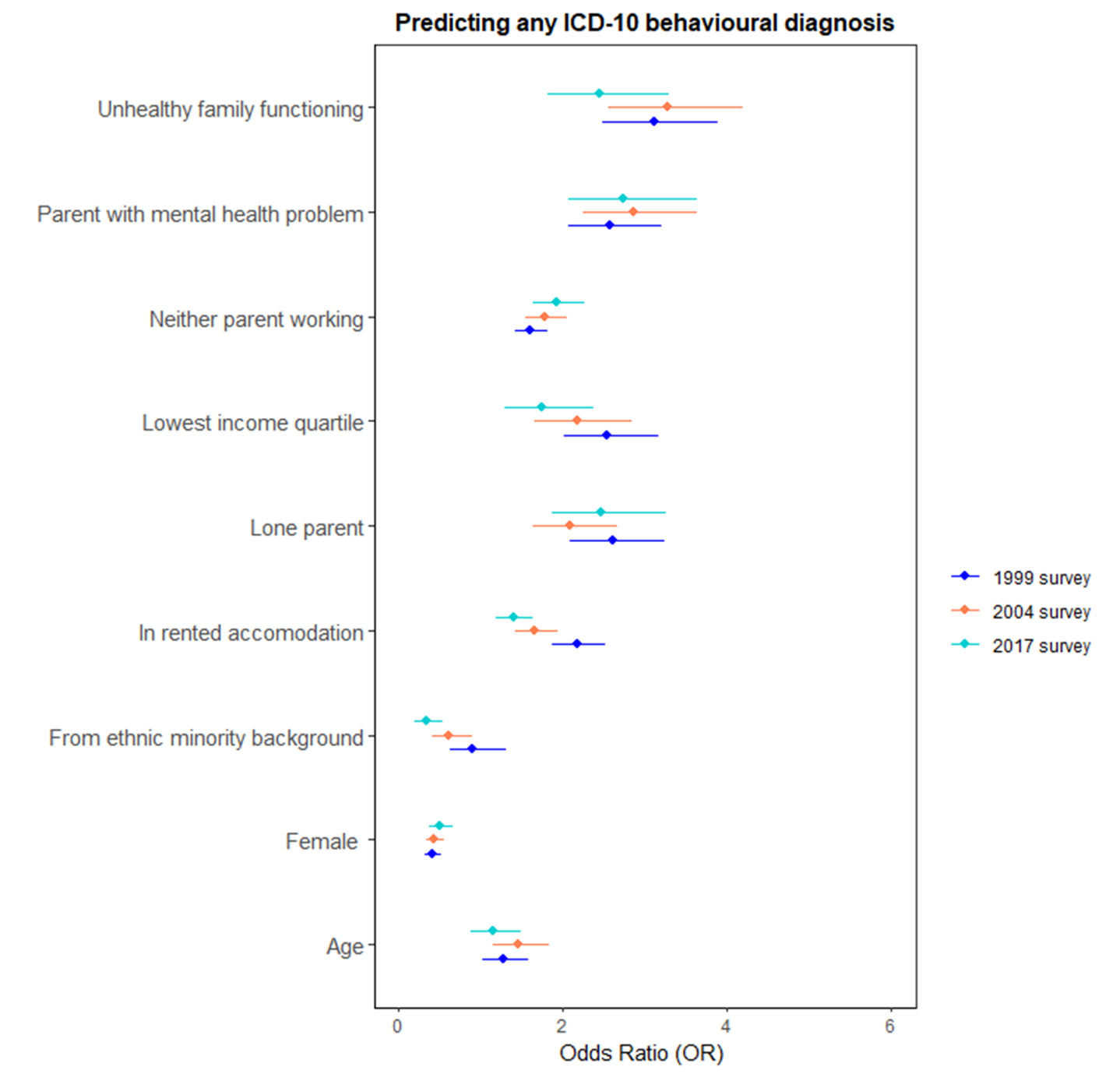


**Figure S7**: Odds of children having any hyperkinetic disorder based on sociodemographic and family characteristics


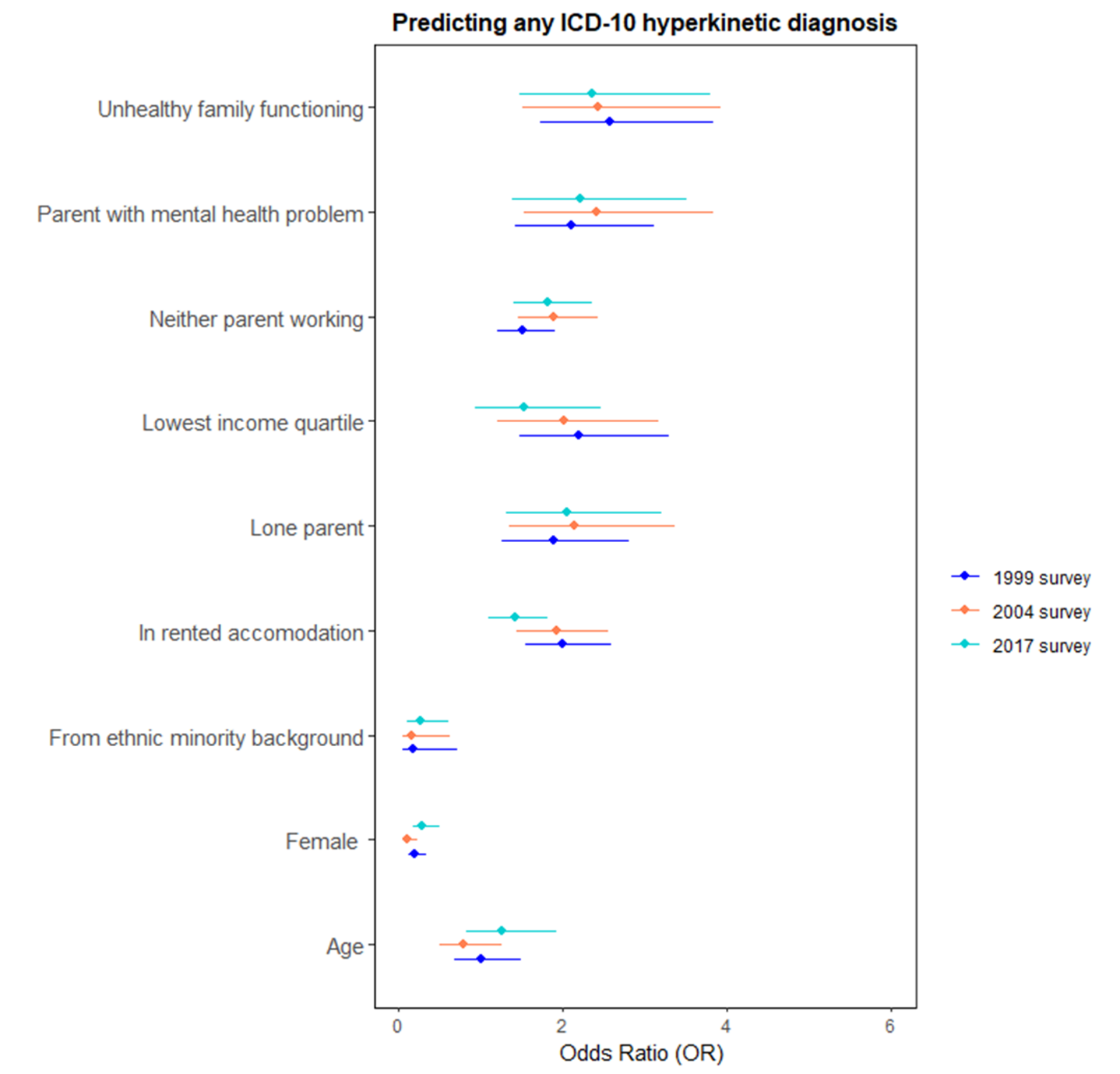


**Figure S8**: Odds of children having any comorbid disorder based on sociodemographic and family characteristics


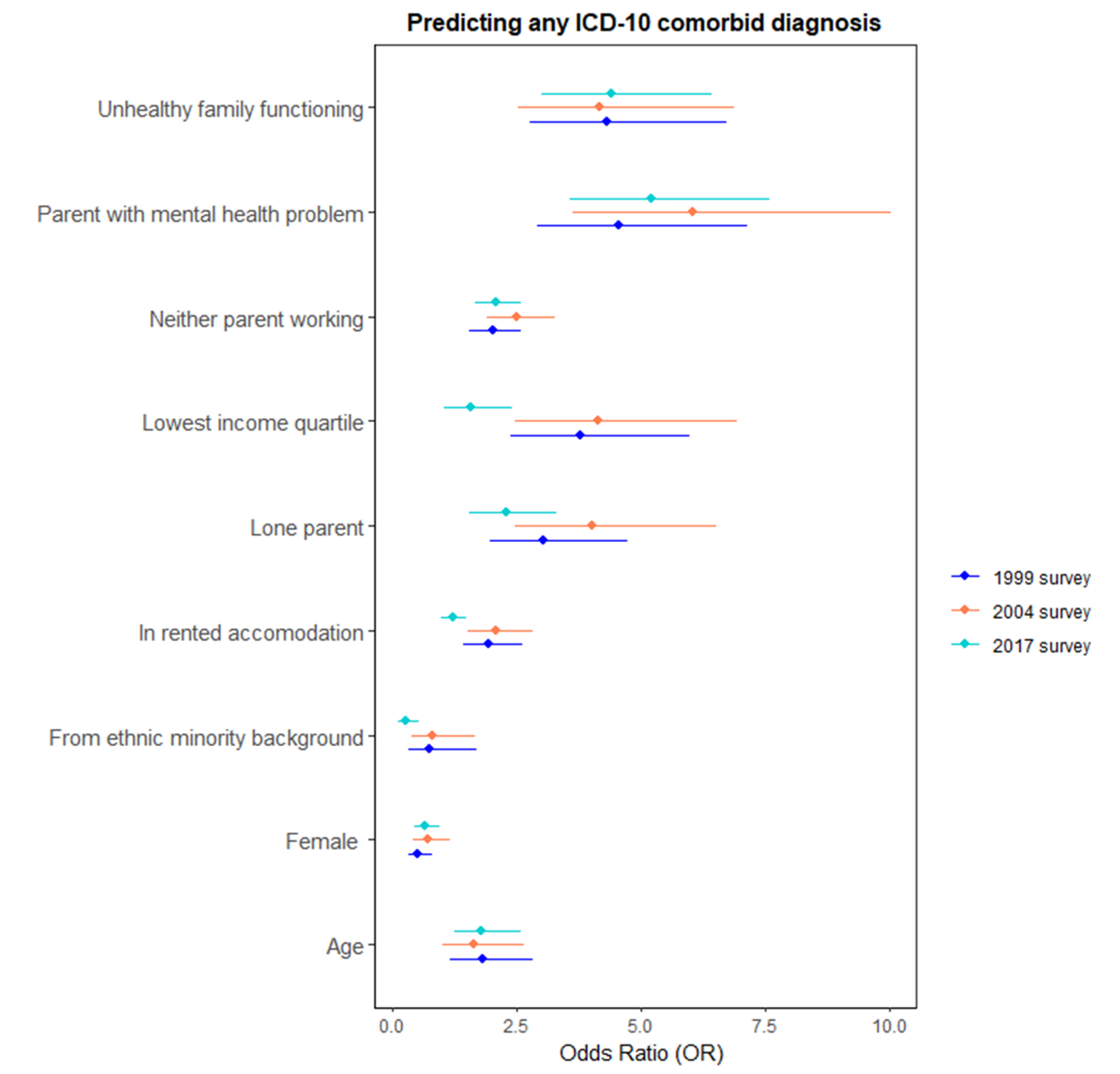

Supplement: Supplementary file 1 — Table S1. Survey sample sizes and missing data (England only). Table S2. Survey sample baseline characteristics (England only). Table S3. Comparison of ethnicity of children with any ICD‐10 disorder across surveys. Table S4. Comparison of difficulties of children (5–10 years) and adolescents (11–15 years) with any ICD‐10 disorder. Table S5. Comparison of difficulties of those aged 5–15 years with any ICD‐10 disorder, stratified by ethnicity. Table S6. Comparison of impact of those aged 5–15 years with any ICD‐10 disorder, stratified by ethnicity. Table S7. SDQ total difficulty scores in 1999, 2004 and 2017, among 5–15 year olds with a DAWBA‐identified psychiatric disorder (England only), controlling for entropy balanced weights. Table S8. Comparison of difficulties of children (5–10 years) and adolescents (11–15 years) with any ICD‐10 emotional disorder. Table S9. Comparison of difficulties of children (5–10 years) and adolescents (11–15 years) with any ICD‐10 behavioural disorder. Table S10. Comparison of difficulties of children (5–10 years) and adolescents (11–15 years) with any ICD‐10 hyperkinetic disorder. Table S11. Comparison of difficulties of children (5–10 years) and adolescents (11–15 years) with any ICD‐10 comorbid disorder. Table 12. Comparison of sociodemographic and family characteristics of children with DAWBA‐identified psychiatric disorder (univariable models). Table S13. Comparison of characteristics of children with any emotional disorder (all ages). Table S14. Comparison of characteristics of children with any emotional disorder (11–15 years). Table S15. Comparison of characteristics of children with any behavioural disorder (all ages). Table S16. Comparison of characteristics of children with any hyperkinetic disorder (all ages). Table S17. Comparison of characteristics of children with any cross‐comorbid disorder (all ages). Figure S1. Cross survey comparison of parent‐ and teacher‐rated mean total difficulty scores (SDQ) among those aged 5–15 [file JCPP-66-167-s001.docx]
